# Active control of strong plasmon–exciton coupling in biomimetic pigment–polymer antenna complexes grown by surface-initiated polymerisation from gold nanostructures†

**DOI:** 10.1039/d1sc05842h

**Published:** 2022-02-03

**Authors:** Anna Lishchuk, Evelin Csányi, Brice Darroch, Chloe Wilson, Alexei Nabok, Graham J. Leggett

**Affiliations:** Department of Chemistry, University of Sheffield Brook Hill Sheffield S3 7HF UK Graham.Leggett@sheffield.ac.uk; Materials and Engineering Research Institute, Sheffield Hallam University City Campus Sheffield S1 1WB UK

## Abstract

Plexcitonic antenna complexes, inspired by photosynthetic light-harvesting complexes, are formed by attachment of chlorophylls (Chl) to poly(cysteine methacrylate) (PCysMA) scaffolds grown by atom-transfer radical polymerisation from gold nanostructure arrays. In these pigment–polymer antenna complexes, localised surface plasmon resonances on gold nanostructures are strongly coupled to Chl excitons, yielding hybrid light–matter states (plexcitons) that are manifested in splitting of the plasmon band. Modelling of the extinction spectra of these systems using a simple coupled oscillator model indicates that their coupling energies are up to twice as large as those measured for LHCs from plants and bacteria. Coupling energies are correlated with the exciton density in the grafted polymer layer, consistent with the collective nature of strong plasmon–exciton coupling. Steric hindrance in fully-dense PCysMA brushes limits binding of bulky chlorophylls, but the chlorophyll concentration can be increased to ∼2 M, exceeding that in biological light-harvesting complexes, by controlling the grafting density and polymerisation time. Moreover, synthetic plexcitonic antenna complexes display pH- and temperature-responsiveness, facilitating active control of plasmon–exciton coupling. Because of the wide range of compatible polymer chemistries and the mild reaction conditions, plexcitonic antenna complexes may offer a versatile route to programmable molecular photonic materials.

## Abstract 2

Excitons in pigment–polymer antenna complexes formed by attachment of chlorophyll to surface grafted polymers are coupled strongly to plasmon modes, with coupling energies twice those for biological light-harvesting complexes and active control of plasmon–exciton coupling.

## Graphical abstract

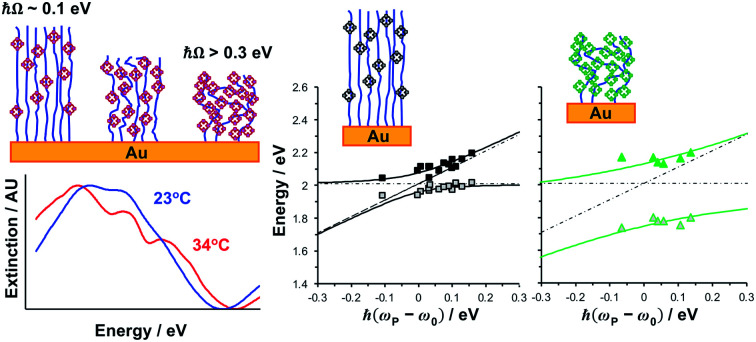

## Introduction

The absorption of light by molecules leads to the formation of excitons (electron–hole pairs). Exciton dynamics in molecular systems are typically dominated by incoherent dipole coupling mechanisms;^1,2^ consequently, exciton diffusion lengths are of the order of 10 nm, or in exceptional cases a few tens of nm,^2^ significantly constraining the design of devices.^1,2^ Thus, an unsolved grand challenge has been to develop design rules for the efficient long-range transport of excitons in molecular materials.

There has been intense interest in the possibility that photosynthetic light-harvesting antenna complexes (LHCs) from plants and bacteria may provide a model for the efficient transfer of excitons in molecular systems.^3–9^ In these pigment–protein complexes, peptide units organise pigment molecules (chlorophylls and carotenoids) precisely in space, regulating networks of energy transfer steps with remarkable precision.^10^ However, proteins are not well-suited for putative applications of photonic materials, because of the high cost of large-scale manufacturing and their intrinsic susceptibility to damage during processing. Thus, to incorporate biologically-inspired design principles into photonic devices, for example in the development of novel light-harvesting systems and biological sensors, new synthetic materials are required that embody the desirable characteristics found in LHCs.

Here, we explore the feasibility of constructing pigment–polymer antenna complexes, biomimetic systems in which the peptide scaffold in LHCs is replaced by a surface-grafted polymer. This is a formidable challenge, because the antenna complexes in photosynthetic systems are highly optimised; they achieve very high pigment packing densities, while also preventing concentration-quenching.^11,12^ Moreover, light-harvesting systems respond dynamically to external stimuli.^13^ For example, nonphotochemical quenching^14–18^ can be switched on under high illumination levels to dissipate energy safely.

One possible approach to the design of biomimetic pigment–polymer antenna complexes is to use polymers formed by surface-initiated atom-transfer radical polymerisation (ATRP)^19–21^ as scaffolds to support pigment molecules. ATRP is a form of living radical polymerisation that yields thick, uniform polymer films with low termination rates and good control of polymerisation kinetics. In activators regenerated by electron transfer (ARGET) ATRP,^22–24^ it is possible to achieve rapid synthesis of grafted polymer films with thicknesses that are controlled *via* modification of the grafting density and polymerisation time. The grafting density is controlled by varying the density of initiators (*e.g.* Br or Cl).^25^ At low initiator densities, surface-grafted polymer molecules are collapsed, forming mushrooms.^25,26^ However, the grafting density can be increased by increasing the initiator density. At high enough densities, steric repulsion occurs between neighbouring polymer chains and brushes are formed.^25,26^ Moreover, stimulus-responsive polymers can be designed that switch their conformational states in response to changes in their local environment.^21,26,27^

There is extensive literature on the synthesis of dye-functionalised polymer brushes,^28–37^ but predominantly work has focussed on the design of polymers with low concentrations of dye molecules. To create biomimetic pigment–polymer antenna complexes, it is instead necessary to achieve very high densities of pigment binding: chlorophyll (Chl) concentrations of ∼0.6 M are found in plant light-harvesting complexes. This places different constraints on the synthetic approach.^12^ Here we describe the synthesis of poly(cysteine methacrylate) (PCysMA)^38^ scaffolds in which the Chl density is regulated by controlling the grafting density and polymerisation time during ARGET-ATRP.

To achieve efficient, ultra-fast transport of energy in synthetic pigment–polymer antenna complexes, we additionally incorporate strong plasmon–exciton coupling. Localised surface plasmon resonances (LSPRs) are formed by resonant coupling of light to the plasmon modes of sub-wavelength metallic nanostructures, and have been widely used in applications ranging from sensors to fundamental studies of LHCs.^39–45^ Under certain conditions, an ensemble of excitons and a plasmon mode supported at a nanoparticle surface may exchange energy faster than their respective decay channels, enabling the system to enter the strong coupling regime in which new hybrid light–matter states (plexcitons) are formed *via* a linear combination of the plasmon and exciton states.^46^ These new states are manifest as a splitting of the plasmon band in the extinction spectrum. Strong coupling has been reported in a variety of types of system, including ones based on dye molecules,^47–49^ J-aggregates^50–58^ and quantum dots.^59^ An important feature of strong plasmon–exciton coupling is that the confined optical mode couples collectively to an ensemble of excitons; thus, plexcitons may be considered as delocalised excited states^60,61^ in which emitters attached to a given nanoparticle are able to exchange energy coherently *via* the light field.^46,60–63^ Theory indicates that exciton diffusion lengths could be enhanced by several orders of magnitude in the strong coupling regime.^46,62^

In previous work, we showed that antenna complexes from bacteria^64^ and plants^65^ are strongly coupled to LSPRs on arrays of gold nanostructures, and showed that the coupling energy could be manipulated *via* control of the protein structure and pigment complement. The collective nature of strong light–matter coupling in these systems was demonstrated in two ways. First, the coupling energy was found to be proportional to the square root of the density of excitons.^64^ Second, in synthetic maquette proteins containing two chlorins, the plasmon mode coupled to a dimer state even though the pigment molecules were too far apart for excitonic coupling through space.^66^

Here, we show that such strong coupling can be replicated using pigment–polymer antenna complexes. Indeed, couplings larger than those observed for photosynthetic LHCs are achieved *via* control of the grafted polymer architecture to yield exciton densities higher than those found in LHCs. Moreover, active control of strong plasmon–exciton coupling is achieved by utilisation of the stimulus-responsive swelling of the polymer scaffold, to yield biomimetic excitation transfer networks that display a high degree of programmability.

## Results and discussion

**Fig. 1:**
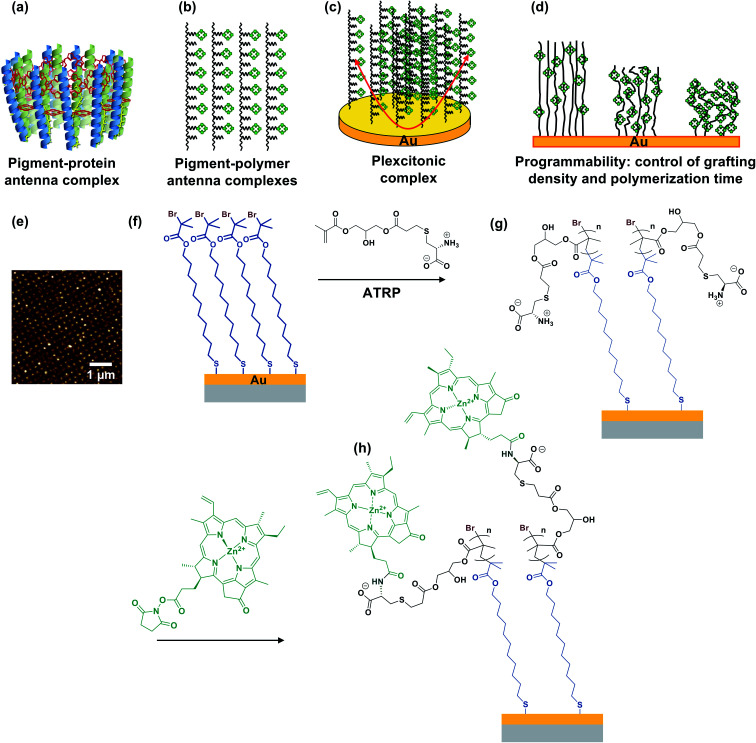
(a) In biological photosynthesis, pigment–protein antenna complexes such as bacterial light-harvesting complex 2 (represented schematically here) regulate energy transfer. Pigments (chlorophylls, red, and carotenoids, yellow) are organised by peptide scaffolds (blue, green). (b) In pigment–polymer antenna complexes, the peptide scaffold is replaced by a surface-grafted poly(amino acid methacrylate), to which chlorophylls are covalently bound after formation of the scaffold. (c) Plexcitonic antenna complexes are formed by strong coupling of excitons in pigment–polymer antenna complexes to confined optical modes. (d) The properties of the plexcitons are programmed by controlling the polymerisation time (molecular weight), grafting density and derivatisation of grafted polymers. (e) Arrays of gold nanostructures formed by interferometric lithography are functionalised with monolayers of initiator-terminated adsorbates (f), enabling growth of poly(cysteine methacrylate) brushes *via* atom-transfer radical polymerisation (g). Attachment of *N*-hydroxysuccinimidyl ester derivatives of chlorophyll a (h) yields plexcitonic antenna complexes.

In photosynthesis, pigment–protein light-harvesting complexes (LHCs, Fig. 1a) organise pigment molecules (chlorophylls and carotenoids) precisely in space using peptide scaffolds.^10^ Here, we describe the synthesis of pigment–polymer antenna complexes, in which surface-grafted poly(amino acid methacrylate) scaffolds replace the polypeptides in LHCs (Fig. 1b). By grafting these pigment–polymer complexes to gold nanostructures, we form plexcitonic complexes *via* strong coupling of localised surface plasmon resonances to the ensemble of excitons arrayed on the polymer scaffolds (Fig. 1c). Strong coupling facilitates ultra-fast transfer of excitation between pigments, and the properties of the coupled modes are programmed *via* modulation of the molecular weight, grafting density and chlorophyll concentration, which control the 3D organisation of excitons within the plasmon mode volume (Fig. 1d). Moreover, active control of strong light–matter coupling can additionally be programmed by exploiting the stimulus-responsive character of the polymer scaffold to change the organisation of excitons within the light field.

Fig. 1e–h show schematically the construction of plexcitonic antenna complexes. A self-assembled monolayer (SAM) of bis[2-(2-bromoisobutyryloxy)undecyl]disulfide is first formed by adsorption from dilute solution onto gold nanostructures (Fig. 1e) formed over ∼0.5 cm^2^ using interferometric lithography.^67^ The disulfide linkage in the adsorbate is cleaved following adsorption to yield a monolayer of bromoisobutyryl-functionalised undecanethiolate (BIBUDT) (Fig. 1f), presenting bromine initiators to the polymerisation solution. Finally, chlorophyll a (Chl) is isolated from spinach and derivatised to introduce *N*-hydroxysuccinimidyl ester pendant groups which react with amine groups on CysMA repeat units to yield a pigment-decorated polymer scaffold (Fig. 1h).

The polymer grafting density is controlled here by co-adsorbing BIBUDT (initiator) with an inert diluent (undecanethiol or 11-mercapto-1-undecanol; both are equally inert to the polymerisation solution) to form a mixed self-assembled monolayer. Isolated surface-grafted polymer molecules formed at low initiator densities are collapsed, forming mushrooms.^25,26^ As the initiator density is increased, the grafting density increases until at high enough densities, steric repulsion occurs between neighbouring polymer chains and brushes are formed.^25,26^ When these densely grafted films are immersed in a good solvent (*e.g.* water for PCysMA), solvent molecules bind to the grafted polymers, creating steric repulsion between neighbouring chains that causes them to repel each other, leading to swelling of the polymer film and an increase in its thickness. We hypothesised that the reactivity of pendant amine groups in PCysMA could be regulated by controlling the polymer grafting density: the lower the grafting density, the lower the steric barrier to penetration of the brush layer by bulky Chl.

### Growth and characterisation of polymer scaffolds

Due to their near-field character, the light fields associated with localised surface plasmon polaritons are confined to sub-wavelength volumes. For gold nanostructure arrays similar to those used here, we previously calculated that the plasmon mode extends 10–30 nm from the metal surface.^64^ Thus, to ensure that effective control of the organisation of pigments within the mode volume is achieved, we first characterised the polymerisation kinetics and solvent interactions to determine the conditions required to achieve control of polymer structure up to 30 nm from the gold substrate.

**Fig. 2:**
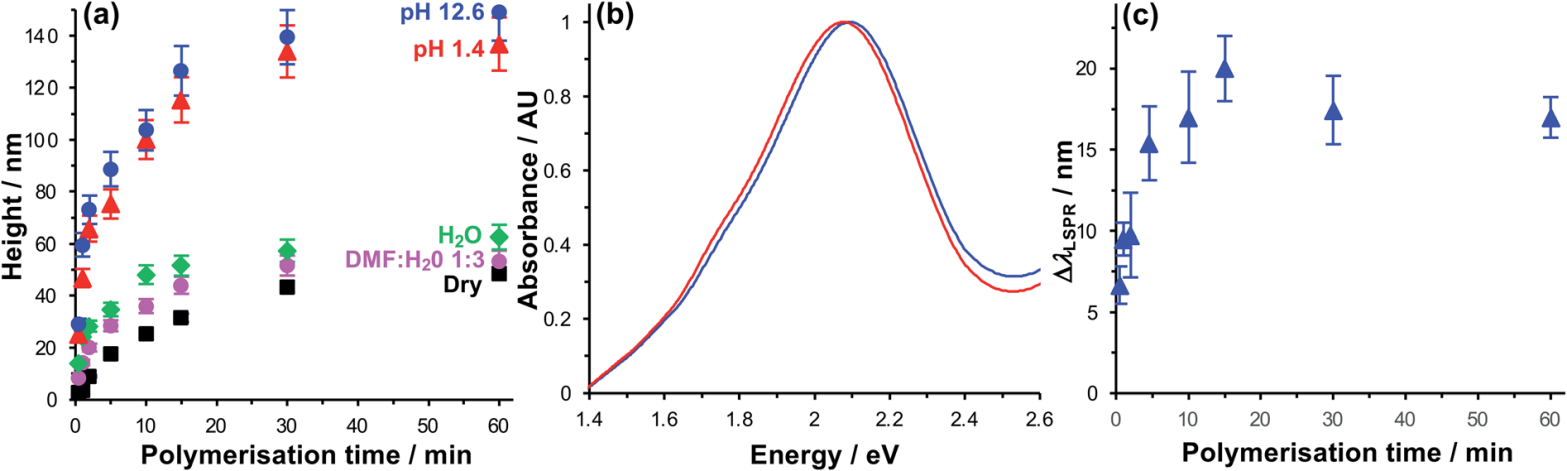
(a) Thickness of fully dense PCysMA films grown from monolayers of BIBUDT adsorbed on planar (polycrystalline) gold films as a function of the polymerisation time, measured using spectroscopic ellipsometry in a variety of media. (b) Extinction spectra of a gold nanostructure array before (blue spectrum) and after (red) growth of a fully dense PCysMA film for 10 min. (c) Red shift in the plasmon band Δ*λ*_LSPR_ for gold nanostructure arrays after growth of fully dense PCysMA films.

The polymerisation kinetics of fully-dense polymer layers (*i.e.* layers grown from complete BIBUDT initiator monolayers) were characterised using spectroscopic ellipsometry (SE). Fig. 2a shows the increase in polymer thickness with polymerisation time *t* for films of PCysMA grown from BIBUDT-functionalised planar gold surfaces. The dry polymer film thickness (black squares) increases monotonically as a function of *t* up to 15 min. This controlled growth of polymer film thickness with time is characteristic of polymerisation by ATRP. After 15 min, chain termination starts to become significant, and the rate of polymer film growth slows. There was little increase in the film thickness for *t* > 30 min.

In dry grafted films, the polymer molecules are collapsed. For *t* = 5 min the dry thickness of the fully-dense PCysMA film is 17.6 ± 0.9 nm (Fig. 2a); thus, in the absence of a solvent the polymer would be expected to extend throughout a substantial fraction of the mode volume. For *t* = 15 min, the film thickness is 31.5 ± 1.6 nm (Fig. 2a); hence the polymer film would be expected to fill the plasmon mode volume when grown from an array of gold nanostructures. Thus, control of the polymerisation time enables control of the composition of the material that lies within the plasmon mode volume.

For extensive Chl-polymer binding, it is necessary that the coupling reaction is carried out in a good solvent for the polymer, so that it is in a swollen conformation. Water is a good solvent for PCysMA; in water, the polymer becomes solvated and PCysMA brushes swell away from the solid surface. For *t* = 15 min, the polymer film thickness in water is 51.6 ± 3.9 nm (Fig. 2a), and the density of polymeric material within the near-surface region is reduced proportionately. However, Chl has limited solubility in water, and is more soluble in 1 : 3 DMF : water mixtures. Thus, we compared the swelling behaviour of PCysMA in 1 : 3 DMF : water mixtures with that in water. The data in Fig. 2a indicate that the swelling of the polymer layer in 1 : 3 DMF–water mixtures is similar to that in water, meaning that this is a good solvent mixture for coupling of Chl to PCysMA brushes.

Under acidic and basic conditions, the zwitterionic monomeric units become, respectively, cationic and anionic, causing additional electrostatic repulsion that causes further substantial swelling of the polymer molecules away from the substrate. Thus, the thickness measured by ellipsometry increases to 115.3 ± 8.6 nm at pH 1 and to 126.5 ± 9.5 nm at pH 12. This stimulus-responsiveness of PCysMA provides a mechanism for active control of light–matter coupling, by exploiting controlled polymer swelling to modulate the density of excitons in the plasmon mode volume (see below).

Extinction spectra were acquired for gold nanostructure arrays before and after growth of PCysMA (Fig. 2b). Growth of the polymer film yielded a small red shift in the position of the plasmon band, as expected, because of the change in the local refractive index close to the gold surface. The red shift increased in size as the polymerisation time increased (red circles in Fig. 2c), and the rate of growth of the red shift was correlated with the rate of growth of the polymer film thickness measured for planar gold films in Fig. 2a.

**Fig. 3:**
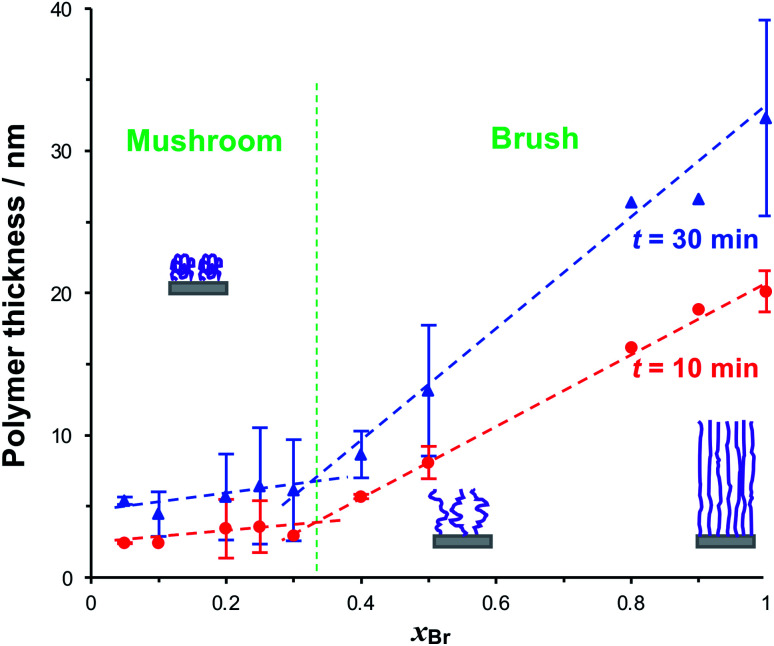
Variation in the dry thickness of grafted polymer layers after polymerisation times of 10 and 30 min as a function of the mole fraction of BIBUDT *x*_Br_ in the thiol solution from which the bromo-functionalised self-assembled monolayer was formed prior to carrying out ATRP. Schematic diagrams represent the three broad conformational groups studied here: full-density brushes (high *x*_Br_), reduced-density brushes (intermediate *x*_Br_), and mushrooms (*x*_Br_ ≤ 0.3). Lines are fitted to the data by linear regression.

To form reduced density grafted films, initiator-bearing monolayers were formed by co-adsorption of bromo-functionalised initiators and a diluent from a solution of the two adsorbates in ethanol with a mole fraction *x*_Br_ of the initiator-terminated thiol. PCysMA films were grown from these surfaces and characterised by SE (Fig. 3). Two distinct regimes may be observed: for *x*_Br_ ≥ 0.4, the grafted polymer thickness increases comparatively steeply as a function of *x*_Br_, while for *x*_Br_ ≤ 0.3, the changes in *x*_Br_ yield much smaller changes in thickness. For *x*_Br_ ≥ 0.4, the grafted polymers are closely packed and form brushes, while for *x*_Br_ ≤ 0.3 a mushroom conformation is adopted, consistent with well-established models of polymer growth by surface-initiated ATRP. For example, Genzer and co-workers^25^ showed that in the brush regime, the height of the grafted layer is given by *H* ∝ *nσ*^0^, where *σ* is the grafting density and *n* is the degree of polymerisation, while in the mushroom regime, *H* ∝ *nσ*^1/3^.

**Fig. 4:**
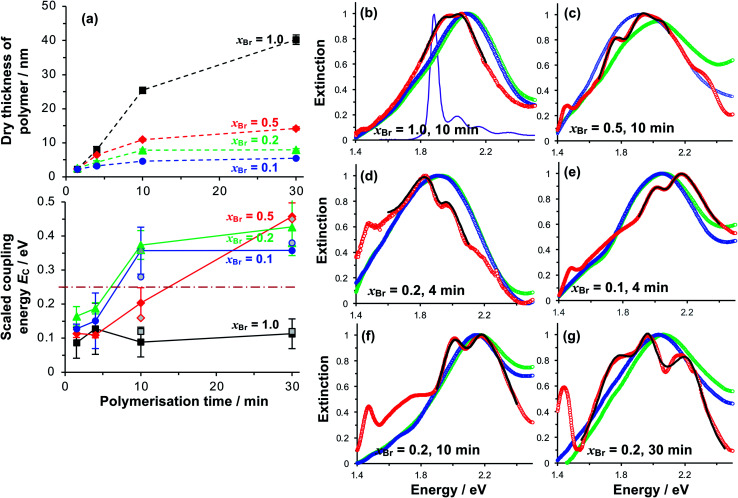
(a) Variation in the polymer dry thickness and scaled coupling energy *E*_C_ with polymerisation time for initiator compositions *x*_Br_ = 1.0 (squares), 0.5 (diamonds), 0.2 (triangles) and 0.1 (circles). The horizontal dashed line in the lower graph indicates the threshold for strong plasmon–exciton coupling, 0.25 eV. The error bars are the standard deviations of at least three different measurements for each point. Open symbols for *t* = 10 and 30 min correspond to Rabi energies determined from analysis of the dependence of the energies of the coupled states on the detuning (see also Fig. 5). (b–g) Normalised extinction spectra for nanostructure arrays before (green) and after (blue) growth of grafted PCysMA layers by ATRP with varying initiator density x_Br_. Extinction spectra acquired after binding of Chl are shown in red. Fits to the region around the LSPR are shown as bold black lines. The absorption spectrum for Chl in solution is shown in purple in (b).

We selected three values of *x*_Br_ for further investigation: *x*_Br_ = 0.5, which Fig. 3 indicates should yield reduced-density brushes close to the brush-to-mushroom transition; and *x*_Br_ = 0.2 and 0.1, both of which should yield mushrooms. In reduced-density brushes we expect that steric hindrance to the bulky Chl derivatives will be reduced relative to full-density brushes, enabling more extensive reaction between Chl derivatives and the PCysMA scaffold. A further substantial reduction in steric hindrance is expected for mushrooms, enabling more complete functionalisation of the grafted polymer chains. Polymerisation kinetics for these systems were characterised by SE on planar gold surfaces. Data are shown in Fig. 4a (top). After 30 min, the thickness of the fully-dense film was 40.3 nm, while for films grown from initiator layers with *x*_Br_ = 0.5, 0.2 and 0.1, we measured thicknesses of 14.2, 7.9 and 5.5 nm respectively.

### Extinction spectra of plexcitonic complexes

Extinction spectra of plexcitonic complexes are shown in Fig. 4b–g. The spectra of the clean nanostructure arrays are coloured green; spectra acquired after PCysMA film growth are coloured blue; and spectra acquired after attachment of Chl are red. Growth of the grafted polymer layer led to a small red shift in the position of the plasmon band, attributed to the change in the local refractive index from air to that of the polymer (*n* = 1.53), but no other significant changes were observed. However, attachment of Chl caused splitting of the plasmon band that was attributed to plasmon–exciton coupling and was correlated with changes in the polymer grafting density. For fully-dense brushes, this splitting was small (Fig. 4b), and the splitting did not change as the polymerisation time increased, suggesting that the density of excitons in the plasmon mode volume remained constant.

For reduced density brushes, significant changes were observed after coupling of Chl to polymer scaffolds. For *x*_Br_ = 0.5 and polymerisation time *t* < 10 min, a modest splitting of the plasmon band was observed, similar to that in Fig. 4b. However, when *t* was increased to 10 min, the splitting was increased after attachment of Chl (Fig. 4c). Additionally, a new feature is also evident at 1.46 eV in Fig. 4c. A peak is observed at this energy in extinction spectra of clean nanostructure arrays, but at greatly reduced intensity; thus, it does not correspond to a plexcitonic state. In previous studies, the feature at 1.46 eV was found to be enhanced for arrays that were strongly coupled to light-harvesting complexes.^65^

For LHCII, the intensity of this feature increased with the density of nanostructures in the array, suggesting that it may result from enhanced evanescent energy transfer between nanostructures due to strong coupling.^65^ Thus while it is not a plexcitonic state, it appears to be enhanced by plasmon–exciton coupling.

For mushrooms, there was evidence of increased splitting of the plasmon band even after short polymerisation times. For example, after *t* = 4 min, the splitting of the plasmon mode is more pronounced for both *x*_Br_ = 0.2 (Fig. 4d) and for *x*_Br_ = 0.1 (Fig. 4e). The feature at 1.46 eV is pronounced in both of Fig. 4e and f. Furthermore, in both spectra, an additional broad feature is observed between 1.6 and 1.8 eV. This feature is broader than the plasmon band, underlining that it has a different origin than the features formed by splitting of the plasmon band. Pronounced splitting of the plasmon band is observed for longer polymerisation times (*e.g.* 30 min, Fig. 4g). These spectra suggest that control of the polymeric antenna architecture has a profound influence on plasmon–exciton coupling.

### Modelling of plasmon–exciton coupling

To test whether the data in Fig. 4 are consistent with strong plasmon–exciton coupling, the system was modelled as coupled harmonic oscillators, following the approach described in our earlier work on strong coupling to LHCs.^64,66^ As noted above, the features observed at 1.46 and 1.6–1.8 eV are not thought to be attributable to plexciton states. However, the latter feature lies close to the plasmon band and for systems with the strongest coupling, where it was intense, an uncoupled resonance was included in the fitting to account it.

**Fig. 5:**
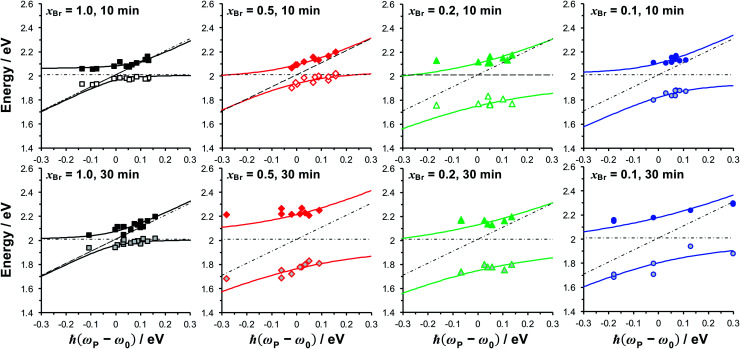
Dependence of the energies of the coupled modes on the detuning for fully dense films (*x*_Br_ = 1.0), reduced density brushes (*x*_Br_ = 0.5) and mushrooms (*x*_Br_ = 0.1, 0.2). The energies of the exciton and plasmon states are given by, respectively, the horizontal and diagonal dashed lines. Solid lines are fitted to the upper and lower plexciton states using eqn (4).

**Fig. 6:**
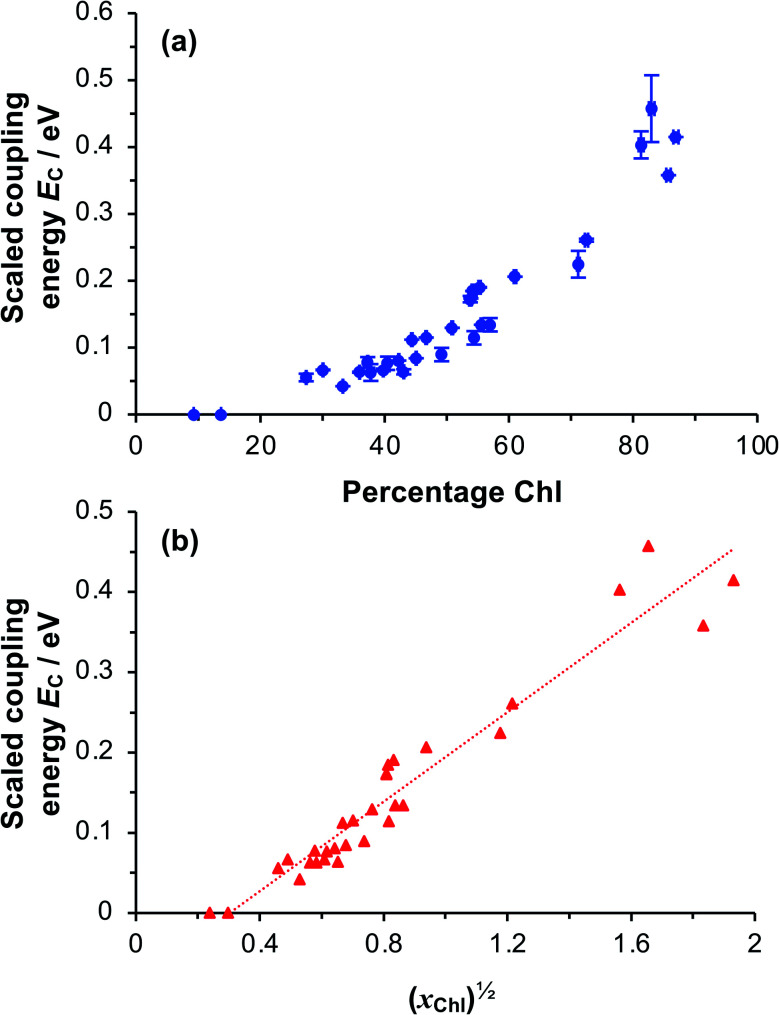
(a) Relationship between the scaled coupling energy and the film composition, as determined using spectroscopic ellipsometry. (b) Relationship between the scaled coupling energy and the square root of *x*_Chl_, the mole fraction of monomers with a bound Chl.

**Fig. 7:**
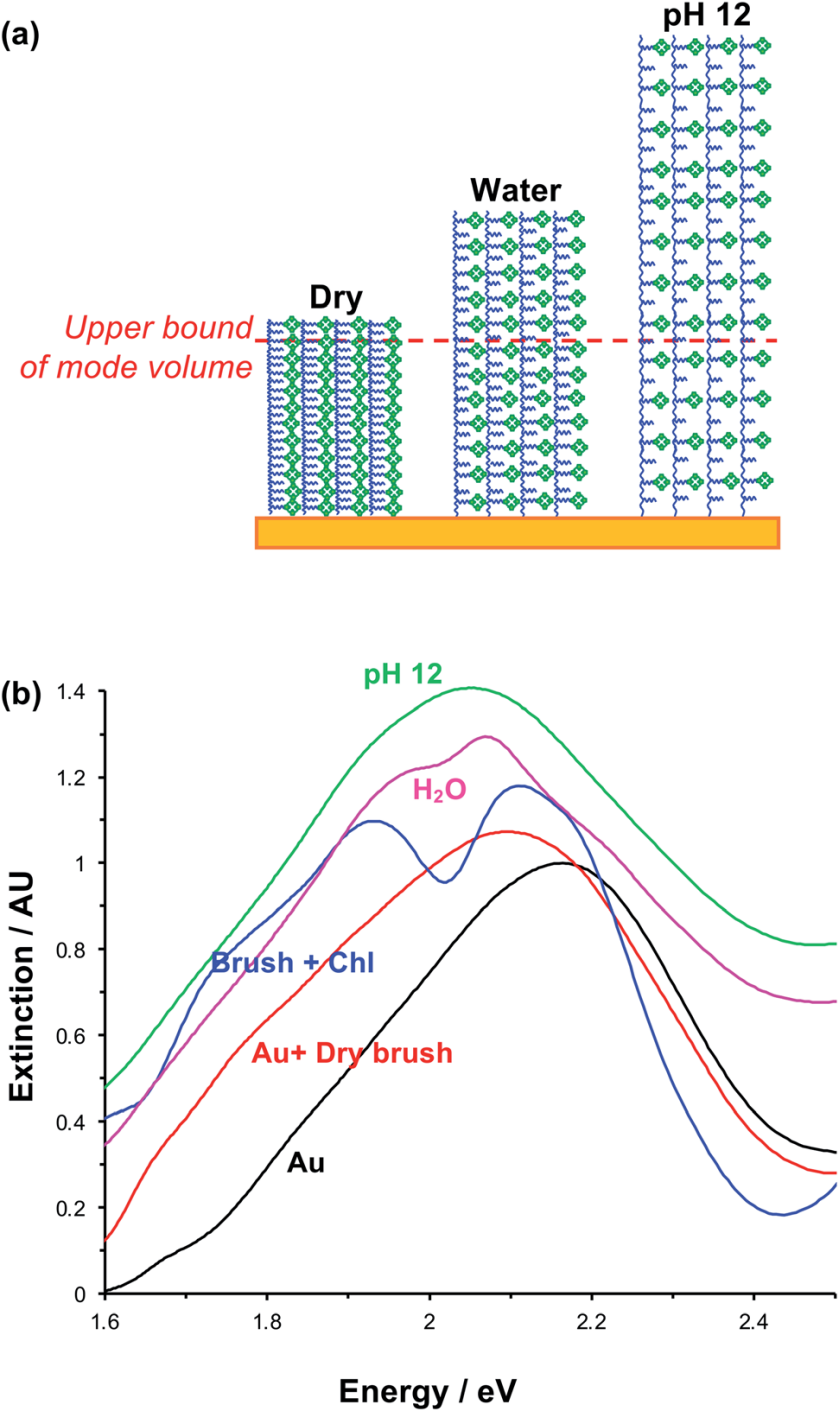
(a) Schematic diagram showing the change in exciton density within the plasmon mode volume that results from swelling associated in the change from dry to hydrated brushes, and from additional electrostatic repulsion between polymer scaffolds at pH 12. (b) Extinction spectra acquired for plexcitonic complexes in different media.

In the coupled oscillator model the coupling constant *g* has the dimension of frequency squared. At resonance when the two oscillators have the same frequency *ω*, the splitting between the normal modes is approximately *g*/*ω*.^45^ For comparison with the experimental data, we here scale the equations to have units of energy rather than frequency; thus, in our model the coupling *G* (for details, see ESI†) has dimensions of energy squared. To connect to microscopic quantum and semi-classical descriptions of strong coupling, we present in Fig. 4a the scaled coupling energy *E*_C_ = *G*/*E*_LSPR_, approximately equal to the splitting in the normal modes when the oscillators are at resonance. Thus, the scaled coupling energy reported in Fig. 5–7 is significantly smaller than the modelled splitting between the plexcitons, *E*_S_, determined by fitting the spectra.

For fully-dense films, the mean scaled coupling energy was found to be ∼0.1 eV for all film thicknesses. For a splitting in the normal modes to be visible, it is necessary for the difference in the energies of the real modes at resonance1
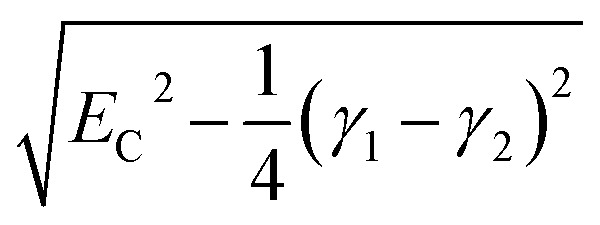
to remain real.^46^ Thus, one criterion for strong coupling is that2
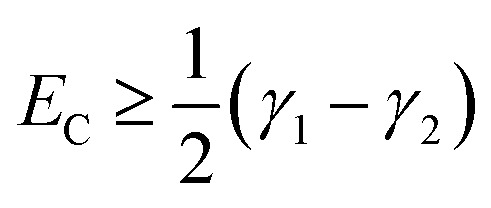
Here, *γ*_LSPR_ ∼ 0.6 eV and *γ*_mol_ ∼ 0.1 eV, hence the splitting should be ≥0.25 eV, a condition that is not satisfied for these full-density brushes. An alternative criterion for strong coupling is that^46^3
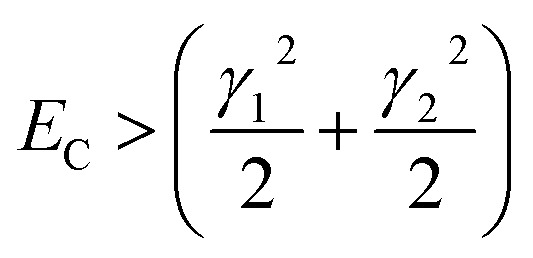


This sets a slightly lower bar of 0.19 eV, but this is not reached for the fully-dense brushes either.

The exciton energy was determined from the fitting to be 2.0 eV, larger than the energy of the Chl Qy peak (1.85 eV). Small features attributed to vibronic coupling are observed to the high energy side of the Qy peak (Fig. 4b); it is possible that the plasmon mode couples instead to one of these. Alternatively, it is possible that the plasmon mode couples to a new state formed under strong coupling, similar to the coupling to chlorin H-dimers reported in our previous study of maquette proteins.^66^ The data available to us at present do not allow a definitive determination. However, the Chl Soret band (2.9 eV) has an energy too high to couple to the gold plasmon mode.

For reduced density brushes (*x*_Br_ = 0.5) at *t* < 10 min, the scaled coupling energy *E*_C_ is similar to that for the fully dense film (Fig. 4a, bottom). However, for mushrooms (*x*_Br_ = 0.2 and *x*_Br_ = 0.1), *E*_C_ is significantly larger, and for these reduced initiator densities the strong coupling limit (dashed horizontal line in Fig. 4a, bottom) is approached after only 4 min polymerisation time. For reduced density films, the Chl concentration is much larger because the steric constraints on Chl attachment are greatly reduced.

At *t* = 10 min, reduced density brushes (*x*_Br_ = 0.5) yield *E*_C_ = 0.20 ± 0.04 eV, approaching the strong coupling limit. However, for mushrooms (*x*_Br_ = 0.2 and *x*_Br_ = 0.1), the scaled coupling energies are increased to 0.37 ± 0.04 eV and 0.36 ± 0.07 eV, respectively, significantly in excess of the threshold for strong plasmon–exciton coupling.

For *t* = 30 min, the coupling energy does not increase further for mushrooms (*x*_Br_ = 0.2 and *x*_Br_ = 0.1). However, for reduced density brushes (*x*_Br_ = 0.5), *E*_C_ increases dramatically to 0.46 ± 0.04 eV. We hypothesise that for *x*_Br_ = 0.5 at *t* > 10 min, some of the grafted chains undergo terminations, leading to a reduction in the chain density and a concomitant increase in reaction derivatisation by Chl. Effectively, the outer portion of the reduced density brush begins to resemble a mushroom morphology; steric hindrance to Chl is reduced and the Chl grafting density is thus greatly increased. At *t* = 30 min, polymer films grown from monolayers with *x*_Br_ = 0.5 are only 15 nm thick, and most, if not all, of the film probably lies within the plasmon mode volume. The chain density is higher than that for *x*_Br_ = 0.2 and *x*_Br_ = 0.1, yet low enough for extensive attachment of Chl; thus, the films produced for 30 min growth and *x*_Br_ = 0.5 yield the highest concentration of excitons within the mode volume and thus the largest value of *E*_C_ (0.46 ± 0.04 eV).

For *x*_Br_ = 0.2 and *x*_Br_ = 0.1 the polymer film thicknesses are only 7.9 and 5.5 nm, respectively, for *t* = 30 min, compared to 40.3 nm for the fully-dense brush (Fig. 2a). Thus, while the degree of polymerisation (*i.e.* the number of repeat units in the polymer chain) is expected to be similar for all films, the polymer molecules in the reduced density films are collapsed and the entire film, including all Chl, lies within the plasmon mode volume. The thicknesses of these films are similar to the thicknesses of the protein monolayers used in our previous studies of strong coupling of plasmon modes to light-harvesting complexes, but the coupling energies are significantly larger, consistent with the presence of higher concentrations of pigment molecules.

In the Jaynes–Cummings model of strong light–matter coupling, the frequencies of the coupled modes are given by^45^4
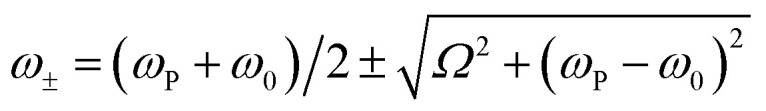
where *ω*_P_ and *ω*_0_ are the frequencies of the plasmon mode and exciton, respectively, and *Ω* is the coupling strength. To further test the hypothesis that our systems displayed strong light–matter coupling, we investigated the dependence of the energies of the coupled modes on the detuning, (*ω*_P_ − *ω*_0_). PCysMA scaffolds were grown from 84 different gold nanostructure arrays with and derivatised by attachment of Chl. Polymerisation times of 10 and 30 min were compared. For each sample, the energies of the coupled modes were determined, and plotted as a function of the detuning (Fig. 5). The solid lines in Fig. 5 show fits to the experimental data using eqn (4), while the horizontal and sloped dashed lines show the exciton and plasmon energies, respectively. An avoided crossing is observed for all polymerisation times and grafting densities. In Fig. 5 the Rabi energy is given by the separation between the curves when *ω*_P_ = *ω*_0_. It can be seen that this energy is small for *x*_Br_ = 1.0, consistent with weak coupling in these films. For *x*_Br_ = 0.5, the coupling is also small for *t* = 10 min. However, on increasing the polymerisation time to 30 min, a dramatic increase in the coupling strength is observed, consistent with the data in Fig. 4. For the mushrooms (*x*_Br_ = 0.2, 0.1), the coupling strength is large for both polymerisation times.

The coupling strengths determined from Fig. 5 are displayed in Fig. 4a (bottom). Within experimental error, estimates of the Rabi energy obtained by the two methods are close. The values determined from Fig. 5 are more rigorous. However, the scaled coupling energy provides a simpler and more convenient measure of coupling strength in experiments that require the comparison of large numbers of samples (see below).

### Concentration dependence of the coupling energy

Because strong plasmon–exciton coupling is a collective phenomenon, the plasmon mode couples to an ensemble of quantum emitters within the plasmon mode volume. The scaled coupling energy is^46^5
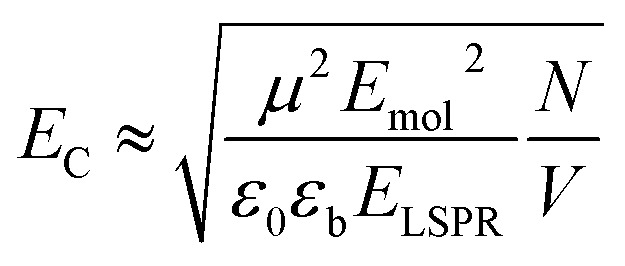
where *N* is the number of excitons in volume *V*, *μ* is the transition dipole moment, *E*_mol_ is the energy of the exciton, *E*_LSPR_ is the energy of the plasmon mode, *ε*_0_ is the permittivity of free space and *ε*_b_ is the relative permittivity of the medium. Thus, we are able to estimate the density of excitons in the mode volume for our plexcitonic complexes.

For Chl a, the transition dipole moment of the Qy transition is estimated to be 5.7 D in a polymer matrix with refractive index *n* ∼ 1.5.^68^ Fitting of the extinction spectra yields an exciton energy of 2 eV, slightly larger than measured for Chl in solution or in polymer scaffolds attached to glass (see ESI†). The relative dielectric constant of PCysMA is not known, but many acrylic polymers have values of ∼3. Thus, *ε*_b_ was estimated as the value for poly(methyl methacrylate), 3.12.^69^ For a plasmon mode with energy ∼2 eV and a scaled coupling energy *E*_C_ ∼ 0.1 eV (fully-dense brushes), we obtain *N*/*V* ∼ 6.1 × 10^25^ m^−3^, slightly less than the exciton density in a monolayer of LH2 (∼8 × 10^25^ m^−3^), consistent with the lower coupling energy. However, for *E*_C_ ∼ 0.37 eV (*x*_Br_ = 0.2, *t* = 30 min) and *E*_C_ ∼ 0.46 eV (*x*_Br_ = 0.5, *t* = 30 min) we obtain exciton densities in the plasmon mode volume of ∼8.4 × 10^26^ m^−3^ and ∼1.3 × 10^27^ m^−3^, respectively, an order of magnitude greater than the exciton density for a monolayer of LH2. For the system displaying the largest coupling energy, the film thickness is 14.2 nm, over twice the thickness of LH2; thus, the exciton density per unit volume of film is equivalent to a concentration of ∼2 mol dm^−3^; by comparison the Chl concentration in plant light harvesting complex II (LHCII) is 0.6 mol dm^−3^.^12^

It might be expected that for such high Chl concentrations, excitonic coupling would lead to significant changes in the energies or linewidths of the excitons. To test this, Chl were attached to grafted polymers grown from glass surfaces with conformations spanning the range from fully dese brushes to mushrooms (see ESI†). However, we did not observe a significant change in either the energy or the linewidth of the Qy peak as a function of the polymer conformation.

To test whether the coupling energy depends on the square root of the Chl concentration as predicted by eqn (5), we first determined the composition of films of pigment–polymer complexes using spectroscopic ellipsometry (SE) (Fig. 6a). Details of the modelling are given in the ESI.† It is clear from Fig. 6a that the scaled coupling energy increases with the Chl content of the films as expected.

Using these data, we calculated the mole fraction of monomers with a bound Chl, *x*_Chl_. This quantity is proportional to the exciton density in the film. If the grafting density is *σ* and the degree of polymerisation is *n*, the density of monomers per unit area is *nσ* and the density of Chl is *nσx*_Chl_. If the Chl composition determined by SE is proportional to the composition by mass, it is related to *x*_Chl_ by:6
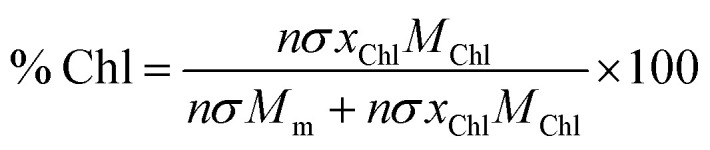
where *M*_Chl_ and *M*_m_ are the relative molecular masses of chlorophyll and the CysMA repeat unit, respectively. Thus, *x*_Chl_ can be estimated, enabling us to plot the scaled coupling energy as a function of (*x*_Chl_)^1/2^ (Fig. 6b). A straight line relationship is observed, indicating that the coupling energy scales with the square root of the exciton density, consistent with eqn (5). It is notable that for those systems that yield the strongest couplings, *x*_Chl_ is greater than unity. In such systems, some Chl must be non-covalently bound, perhaps *via* π–π interactions with covalently bound Chl. Such non-covalent interactions may be important in achieving the high Chl concentrations that are present in plexcitonic complexes formed using mushrooms and reduced density brushes as scaffolds.

### Active control of plasmon–exciton coupling

The swelling of polymer brushes is regulated by the medium (Fig. 2a). Thus, we hypothesised that the density of excitons in the mode volume may be regulated by changing the medium and hence the swelling of the grafted layer (see schematic diagram in Fig. 7a). Extinction spectra are shown for a variety of media in Fig. 7b. The extinction spectrum of a clean nanostructure array is coloured black in Fig. 7b. After growth of a fully-dense PCysMA scaffold, a small red shift (0.08 eV) is observed in the position of the plasmon band (red trace). After addition of Chl, plasmon–exciton coupling causes splitting of the plasmon band (blue). However, on transfer to water, the splitting of the plasmon band is greatly reduced and is only just evident (pink). This is attributed to swelling of the grafted polymer in water, causing it to swell away from the surface, thus yielding a reduction in the density of Chl within the mode volume. The small splitting observed in water is lost completely when the pH is adjusted to 12, because the carboxylic acid groups on the PCysMA chains are deprotonated (*i.e.* negatively charged), creating electrostatic repulsion that causes further swelling so that the exciton density is too low to achieve significant plasmon–exciton coupling.

**Fig. 8:**
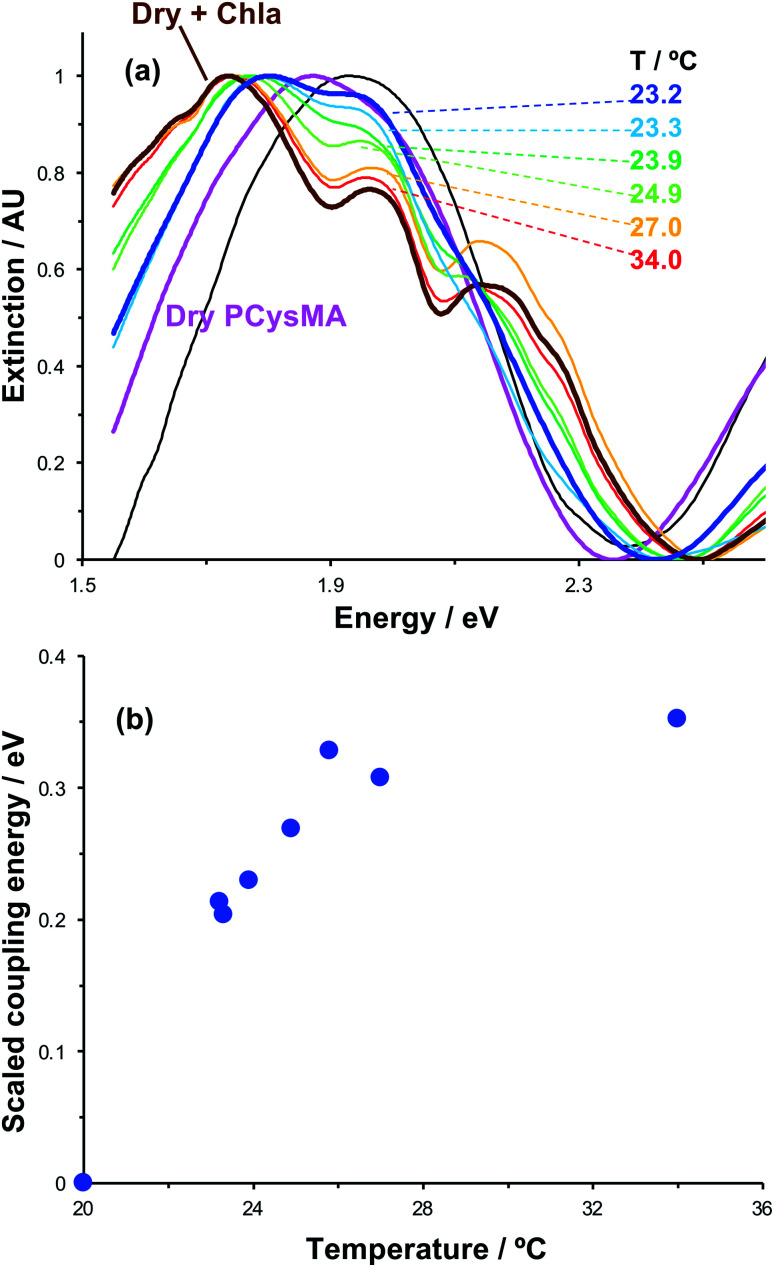
(a) Influence of temperature on plasmon–exciton coupling. A clean gold nanostructure array (black spectrum) is functionalised with a PCysMA scaffold (pink) that is subsequently derivatised by attachment of Chl (brown, bold). On transfer to a 1 : 1 DMF : buffer mixture, the splitting of the plasmon band is greatly reduced (blue, bold spectrum), but progressively restored as the temperature is elevated to 34.0 °C. (b) Variation in the coupling energy *E*_C_ with temperature for plexcitonic complexes immersed in 1 : 1 DMF : buffer mixtures. The errors (standard deviation) are smaller than the symbols used to mark the points.

Active control of strong coupling was also achieved by changing the temperature. In Fig. 8a, growth of a PCysMA layer causes a red shift of 1.87 eV nm in the position of the plasmon band (pink spectrum). Attachment of Chl causes splitting of the plasmon band to yield new peaks at 1.97 and 1.77 eV, attributed to strong plasmon–exciton coupling (brown). However, in a 1 : 1 mixture of dimethyl formamide (DMF) and phosphate buffered saline (PBS), the grafted layer becomes solvated and swells away from the surface, reducing *N*/*V* so that splitting of the plasmon band is not observed. When the temperature is increased to 23.2 °C, weak splitting of the plasmon band emerges, with new features observed at 1.81 eV and 1.95 eV (royal blue). As the temperature is increased, the splitting becomes more pronounced. The low-energy component shifts to progressively lower energies, reaching 1.74 eV at 34 °C. The higher energy component shifts more gradually to 1.97 eV. It is also notable that at temperatures above 25.8 °C a new feature is observed at ∼2.15 eV, close to the energy of the highest energy component in the cluster attributed to Qy/Qx energy transfer in the absorption spectrum of Chl.

We hypothesise that solvation of the grafted layer is decreased at elevated temperatures,^26,27,70^ leading to a temperature-dependent reduction in the thickness of the brush layer; thus, *N*/*V* increases and the system re-enters the strong coupling regime. The system was modelled as coupled harmonic oscillators, and the coupling energies were determined as a function of the temperature (Fig. 8b). For this system, strong coupling is achieved at temperatures above 24.0 °C, and the coupling energy rises to 0.35 ± 0.01 eV at 34.0 °C, when the polymer is fully collapsed. These data provide a powerful demonstration that we can not only program the exciton energy, but also regulate it simply and effectively using a variety of active controls.

### General discussion

Photosynthetic antenna complexes have exquisite structures that organise excitons precisely in three dimensions and control networks of energy transfer steps. Thus, they provide a powerful paradigm for the design of molecular photonic materials. However, for both fundamental investigations of energy transfer in biological systems and for putative technological applications of biologically-inspired materials, it is desirable to be able to construct antenna complexes that are different from those that are found in nature.

One approach, developed by Dutton and co-workers, is to design synthetic pigment–protein complexes;^66,71,72^ however, the challenges of scaling up such a biosynthetic process would be substantial for many putative applications of molecular photonic materials. The present paper illustrates a different approach, in which a synthetic polymer scaffold is used in place of the peptide scaffolds found in many biological light-harvesting complexes. From a synthetic perspective, a primary goal is to achieve pigment densities at least as high as those found in biological light-harvesting complexes. Here, we have demonstrated that exciton densities three times those found in LHCII can be achieved by binding Chl to surface-grafted polymer scaffolds. Moreover, the polymer chemistry involves simple, aqueous processes based upon inexpensive starting materials.

An important aspect of biological light-harvesting systems is their programmability and their responsiveness to external stimuli. For example, purple bacteria are able to adjust the stoichiometry of the membrane components in chromatophore vesicles to optimise ATP production as the light level is changed,^13^ while in plant light-harvesting complexes, non-photochemical quenching provides a mechanism to protect the photosynthetic apparatus when exposed to intense illumination.^11,12,17^ Here, we have shown that by control of the polymer grafting density and the degree of polymerisation, the pigment complement can be changed, and by exploiting the stimulus-responsive character of surface-grafted polymers, it is also possible to adjust the density of excitons in the surface-grafted layer in real time.

An additional intriguing and controversial aspect of the properties of biological light-harvesting complexes is the potential that they may provide for the formation of quantum states that are delocalised across multiple pigment molecules. Two-dimensional electronic spectra of LHCs display signatures that have been attributed to the superposition of excited state eigenfunctions in LHCs to yield “exciton bands”,^3,73–75^ and it has been conjectured that such quantum coherence might enable rapid delocalisation of excitation across multiple pigment molecules.^76^ This has raised hopes that photosynthetic systems may provide design principles for the production of novel biomimetic photonic materials. However, there is now experimental evidence that long-lasting coherence is not found in LHCs, because dephasing is too fast.^77^

Here we present evidence that localised surface plasmon resonances are strongly coupled to excitons in surface-grafted pigment–polymer antenna complexes. An important feature of the resulting plexcitonic states is that they are produced by the collective coupling of an ensemble of excitons to a confined optical mode. Plexcitons may be considered as delocalised excited states^60,61^ in which emitters attached to a given nanoparticle are able to exchange energy coherently *via* the light field.^46,60–63^ As a consequence, in the strong coupling regime, coherent excitation transfer emerges as a necessary consequence of the underlying physics and 
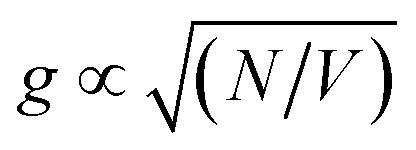
. Thus, while the concept of quantum coherence remains the subject of controversy in biological light-harvesting systems, it becomes a programmable property of plexcitonic antenna complexes because it is facilitated by the underpinning physics of strong light–matter coupling.

It is likely that a wide variety of monomers and dyes would be compatible with the approach described here, which is based on simple, inexpensive, aqueous chemistry and seems to offer a convenient route to the production of programmable photonic materials. Moreover, a variety of polymer systems undergo stimulus-responsive behaviour, creating a large palette of possible materials that incorporate active control of excitation transfer.

There has been growing interest in the active control of plasmon–exciton coupling (for a recent review article, see ref. 77 and 78). The most direct route to active control of strong coupling is *via* alteration of the coupling strength, most readily achieved *via* modulation of the exciton density. We here demonstrated that this is achievable in pigment–polymer complexes *via* alteration of both pH and temperature.

The pH- and temperature-responsiveness of the plexcitonic antenna complexes described here suggests that they may be suitable for adaptation for use as pH- and temperature-sensors; a change in the splitting of an extinction band provides a convenient diagnostic for a specific change in pH or temperature. Further adaptations of the approach, based on the incorporation of different forms of stimulus-responsive behaviour or excitation transfer in the polymer scaffold, are readily envisaged.

## Conclusions

Pigment–polymer antenna complexes are formed by attaching chlorophyll to poly(amino acid methacrylate) scaffolds grown from gold nanostructure arrays. Binding of Chl was correlated with the polymer conformation. For fully-dense brushes, steric hindrance by close-packed polymer chains reduced the extent of reaction between Chl derivatives with active ester linkers and pendant amine groups on the polymer. However, increased levels of Chl derivatisation were measured for reduced-density brushes, and high levels of derivatisation were measured for grafted polymers with mushroom conformations, which present greatly reduced steric barriers to Chl. For these latter systems, very high chlorophyll binding densities are achieved, exceeding those found in natural light-harvesting antenna complexes. When pigment–polymer antenna complexes are grafted to gold nanostructure arrays, their localised surface plasmon resonances are strongly coupled to Chl bound to the polymer scaffold, yielding coupling energies at resonance as large as 0.46 eV. Active control of plasmon–exciton coupling is achieved *via* two different mechanisms. First, changing the solvent enables the use of controllable swelling of the polymer to adjust the exciton density in the mode volume. Second, adjusting the temperature causes changes in polymer swelling and hence controllable modifications to the density of chlorophylls in the grafted polymer layer. The strength of plasmon–exciton coupling can be programmed *via* synergistic control of the polymer grafting density, degree of polymerisation, the medium and the temperature.

## Data availability

Data for this paper, including spreadsheets used to generate Fig. 2–8, are available at the University of Sheffield's Online Data Archive (ORDA) at https://doi.org/10.15131/shef.data.19125446.v1.

## Author contributions

AL carried out all plasmonic measurements and spectroscopic ellipsometry, and modelled the data. EC and BD prepared Chl derivatives and prepared polymer brushes. CW assisted AL in studies of active control of plasmon–exciton coupling. AN contributed to the acquisition of spectroscopic ellipsometry data. GJL conceived and designed the work, interpreted the data and drafted the manuscript.

## Conflicts of interest

There are no conflicts to declare.

## Supplementary Material

SC-013-D1SC05842H-s001
