# Supplementary material for: Active control of strong plasmon–exciton coupling in biomimetic pigment–polymer antenna complexes grown by surface-initiated polymerisation from gold nanostructures
Source: Chem Sci. 2022 Feb 3;13(8):2405–17. doi: 10.1039/d1sc05842h (PMC8864694; doi:10.1039/d1sc05842h)
Supplement: SC-013-D1SC05842H-s001 [file SC-013-D1SC05842H-s001.pdf]

Electronic Supplementary Material (ESI) for Chemical Science.
This journal is © The Royal Society of Chemistry 2022

SUPPLEMENTARY INFORMATION

# Active control of strong plasmon-exciton coupling in biomimetic pigment-polymer antenna complexes grown by surface-initiated polymerisation from gold nanostructures

Anna Lishchuk,[1] Evelin Csányi,[1] Brice Darroch,[1] Chloe Wilson,[1] Alexei Nabok[2] and Graham J. Leggett[1]

*[1]Department of Chemistry, University of Sheffield, Brook Hill, Sheffield S3 7HF, UK;*
*[2]Materials and Engineering Research Institute, Sheffield Hallam University, City Campus, Sheffield, S1 1WB, UK*

## CHEMICALS AND MATERIALS

Acetic acid (≥ 99.7) and ethyl acetate (99.5%) were obtained from Sigma-Aldrich, Poole, UK. Dichloromethane (HPLC grade, 99.8), Dimethylformamide (99%), methanol (HPLC grade, 99.99%), sulfuric acid (≥95%) and sodium hydroxide (≥ 97%) were obtained from Fisher Scientific (Loughborough, UK). Acetone (HPLC grade, 99.8%), ethanol (HPLC grade, ≥ 99.8%) and n-Hexane (HPLC grade, ≥97) were obtained from Honeywell Research Chemicals (Loughborough, UK). Hydrochloric acid (35%) and hydrogen peroxide (30%) were obtained from VWR chemicals (Lutterworth, UK).

Water was deionized and filtered for use with the Elga Purelab Option DV35 water filtration system, measured to the conductivity rating of 15 MΩ $cm^{-1}$.

Microscope coverslip slides and glassware were cleaned by immersion in piranha solution (a mixture of 30% hydrogen peroxide and 70% concentrated sulfuric acid) until the solution stopped bubbling and cooled down to room temperature. The glassware was rinsed thoroughly with deionized water and sonicated for 10 – 15 min before being placed in the oven (ca. 90ºC) to dry before use.

## FABRICATION OF GOLD NANOSTRUCTURE ARRAYS

Microscope coverslip glass slides (22 mm × 50mm, #1.5 thickness) were obtained from Menzel-Gläser, Germany. Gold wire (99.997% trace metals basis, Goodfellow Advanced

Materials, UK) and chromium chips (99.5% trace metals basis, Sigma-Aldrich) were used for the thermal evaporation. 1-Octadecanethiol (98%, Sigma-Aldrich) was used for gold surface functionalization. For the preparation of the gold etchant solution, ammonia solution (32%, Fisher Scientific) and cysteamine (98%, Sigma-Aldrich) were used.

The gold film was deposited on the clean and dry microscope coverslip slides in a diffusion pumped Edwards 306 thermal evaporator, with a bell jar evaporation chamber and a piezoelectric film-thickness monitor. The glass slides positioned on a stage ca. 12 cm above current-driven evaporation sources. Chromium chips were evaporated from a tungsten coiled wire boat, and gold wire was evaporated from a tungsten cup boat. The evaporation chamber was roughed out via a rotary pump to a pressure of $10^{-1}$ mbar, before engaging the diffusion pump for high vacuum. Once a pressure of at least $10^{-6}$ mbar had been reached, the current supplied to the boat was ramped until a deposition rate of 0.1 nm/s had been reached. Chromium adhesive layer was deposited at a rate of 0.1 nm $s^{-1}$ until a thickness of 5 – 7 nm. Gold was deposited at the same rate until a thickness of 20 – 25 nm. After the required thickness was reached, the current was lowered slowly and the Cr/Au coated coverslip slides were left to cool for at least 30 min.

Gold nanostructures were fabricated by interference lithography as described previously.[1] Briefly, we used self-assembled monolayers (SAMs) of 1-octadecanethiol (ODT) as the resist. Immediately after deposition of gold, the glass slides were immersed in 1 mM solution of ODT in degassed ethanol for at least 18 h, to form self-assembled monolayers. The functionalised gold slides were stable for up to one year after SAMs formation. SAMs of ODT on gold were photo-patterned by interferometric lithography (IL). Prior to photolithography, glass slides coated in functionalized gold film were rinsed thoroughly with ethanol, dried under nitrogen, and cut into ca. 0.5 cm × 1 cm samples.

Nanopatterning was carried out in a Lloyd’s mirror two-beam interferometer in conjunction with a laser. UV light (244 nm) from a Coherent Innova 300C frequency-doubled argon-ion laser (Coherent, UK) was passed through a focusing objective, 10 μm pinhole, then a spatial filter and expanded to irradiate a region ca. 0.8 $cm^2$ in size. The angle between the mirror and the sample in the interferometer was varied between 20 ± 2.5º and 30 ± 2.5º. Functionalized gold surfaces were exposed to a dose of 35 – 40 J $cm^{-2}$. A single exposure was used to create a pattern of parallel lines. To create a pattern of dots (diamonds, needles, etc.), a second exposure was carried out with rotation of the sample on the stage by different angles (so-called

Rotation Angle, RA). In order not to overexpose the sample in one direction, the second exposure dose was 15 – 20% lower than the first one.

Photopatterned ODT monolayers on gold were etched by immersion in 2 mM cysteamine with an added 8% v/v of ammonia in ethanol. After etching, the samples were then rinsed with ethanol, dried with nitrogen and annealed in a chamber furnace (Carbolite, UK) at 525 ± 3°C for 120 min. The heating rate was 7°C $min^{-1}$. Strong plasmon bands (in a range from 560 nm to 680 nm, depends on fabrication routine) were observed in extinction spectra after annealing.

An image of a typical plasmonic array of gold nanostructures with finder windows is presented in Fig. S1. Windows offer a simple mechanism for identifying the exact position of points of interest within a sample and allowed us to measure the same area of the plasmonic array during all experimental stages.

Annealed samples were cleaned for re-use by immersion in piranha solution (which was allowed to cool down to room temperature) for 5 – 7 min, washed thoroughly with deionized water and dried under a stream of nitrogen.

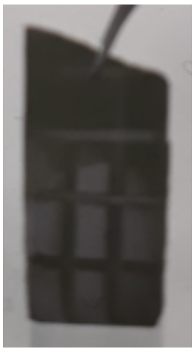

**Figure 1.** Photograph of a typical sample consisting of an array of windows in a finder structure.

*AFM analysis of arrays of gold nanostructures.*

Morphology of the as-fabricated and annealed gold nanostructures was determined with Atomic Force Microscopy (AFM). AFM height images were acquired in the air using a NanoScope Multimode V microscope (Bruker, Germany) operated in a tapping mode. We used OTESPA-R3 model tapping probes (Bruker, Germany) with a resonance frequency of ca. 300 kHz and a nominal tip radius of 7 nm. Image analysis was performed with the Bruker

NanoScope Analysis (v.1.5) software. Morphology and AFM images of typical nanostructures used in this study are presented in Table S1.

**Table S1.** Morphology and AFM images of typical nanostructures.

| | | | |
|---|---|---|---|
| RA=15±2.5°<br>Height=25±2 nm<br>Period=325±5 nm<br>$\lambda_{LSP}$=635±1 nm<br>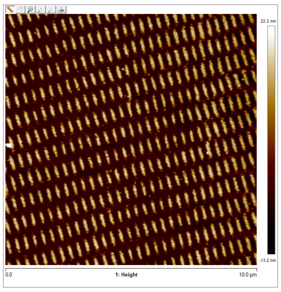 | RA=60±2.5°<br>Height=42±9 nm<br>Period=300±9 nm<br>$\lambda_{LSP}$=592±1 nm<br>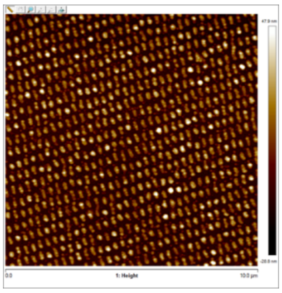 | RA=90°±0.5°<br>Height=38±8 nm<br>Period=295±7 nm<br>$\lambda_{LSP}$=624±1 nm<br>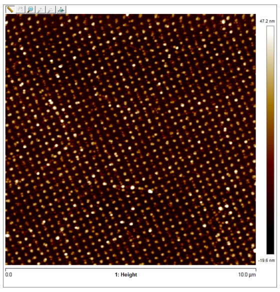 | RA=0°±0.5°<br>Height=25±5 nm<br>Period=250±5 nm<br>$\lambda_{LSP}$=592±1 nm<br>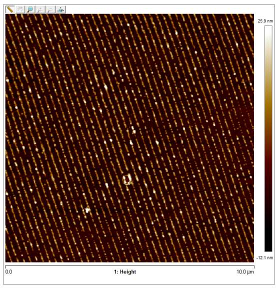 |

SYNTHESIS

*Synthesis of cysteine methacrylate (CysMA)*

The monomer was prepared following the method of Alswieleh et al.[2] L-Cysteine, 3-(acryloyloxy)-2-hydroxypropyl methacrylate, dimethylphenyl phosphine (99%) were obtained from Sigma-Andrich, UK. L-Cysteine (7.54 g, 62.23 mmol) was dissolved in 100 mL of deionised water, and 3-(acryloyloxy)-2-hydroxypropyl methacrylate (13 mL, 14.86 g, 69.36 mmol) was added slowly to the stirred aqueous solution. Afterwards, dimethylphenyl phosphine (20 µL, 1.94 µg, 1.41 x $10^{-8}$ mol) was added as a catalyst to initiate the reaction. The cloudy solution was then stirred at room temperature for 2 h, after which time the solution became colourless. Once complete, the product was washed with ethyl acetate (2 x 100 mL) followed by dichloromethane (3 x 100 mL). The water was then removed by rotary evaporation at 55 – 60°C to produce a white solid. After collection, the solid was then dried under reduced

pressure for 48 h, with analysis confirming the CysMA monomer as a white, crystalline solid (16.175 g, 48.12 mmol, 77%). The resulting CysMA monomer was stored in a desiccator at room temperature.

*Extraction and modification of n-hydroxysuccinimidyl zinc-pyrochlorophyllide a*

2,4,6-collidine (Sigma-Aldrich), dimethylphenylphosphine (DPTS) (99%, Sigma-Aldrich), lithium hydroxide monohydrate (≥98%, Sigma-Aldrich), magnesium sulfate (anhydrous, ≥ 62, 70%, Fisher Scientific), n-(3-dimethylaminopropyl)-*N'*-ethylcarbodiimide hydrochloride (EDC) (Sigma-Aldrich), N-hydroxysuccinimide (NHS) (98%, Sigma-Aldrich), petroleum ether (60-80°C, Sigma-Aldrich), sodium hydrogen carbonate (≥ 99%, Fischer Scientific), tetrahydrofuran (THF) (99.7%, VWR), zinc acetate monohydrate (99.999%, Sigma-Aldrich).

*(i) Extraction of pheophytin **a**.* Spinach leaves (500 g) were procured from a local supermarket retailer. These leaves were prepared by cutting the stems and mid-veins from the leaves and discarding them, followed by washing with deionised water. These were then dried on paper towel and then frozen for 16 h at –20°C. This matter was then macerated in 6 instalments by placing the leaves in a blender with acetone (250 mL) and stirring on a pulse setting for several min until a dark green slurry was obtained. The extract was then filtered by standard vacuum filtration to separate the dark green liquid from the leftover pulp. The acetone and some remaining water were then removed by rotary evaporation to give a dark green substance. This was extracted from remaining water first using petroleum ether 40 - 60°C followed by washing with 60% aqueous methanol. The resulting organic layer was dried over magnesium sulfate, filtered and the solvent removed, leaving a green solid.

To simplify the separation process, all the chlorophylls present in this initial mixture were reduced to their non-metallated pheophytin variants. Here, the solid was dissolved in glacial acetic acid (25 mL) and stirred for 3 h at room temperature. The solution was then neutralised to pH 7 with careful addition of saturated aqueous sodium hydrogen carbonate solution. A brown precipitate was formed in the solution. This precipitate was then extracted from the water layer using dichloromethane and the combined organic layer washed with water before drying and solvent removal as before. The resulting brown solid was then purified by column chromatography (silica, 6:3:2 n- hexane: ethanol: acetone) to give pheophytin *a* as a black solid (235 mg, 0.288 mmol). $R_f$ =0.33 (6:3:1 hexane: ethanol: acetone); LC-TOF-MS ES+, obs. *m/z* = 872 (17.191 min), calc. = 871.22 [$(M + H)^+$, M=$C_{55}H_{74}N_4O_5$].

*(ii) Methyl pyropheophorbide a (Me-PyPh a).* Extracted pheophytin *a* (312 mg, 0.358 mmol) was dissolved in pure 2,4,6-collidine (20 mL) and the vessel backfilled with an argon atmosphere. This solution was then stirred at 130 °C with a reflux condenser under argon for 16 h. Once complete, the collidine was removed using a high-vacuum rotary evaporator (70-80 °C, 1-4 mbar) to give a brown solid. This solid was then immediately dissolved in dichloromethane (20 mL) with addition of sulfuric acid solution (5% in methanol, 20 mL) and stirred at room temperature, again under argon, for another 16 h. The organic mixture was then diluted with dichloromethane (100 mL), washed with water (3 x 100 mL) and once with 10% aqueous sodium bicarbonate (100 mL), followed by drying with magnesium sulfate and solvent removal as before. The resulting solid was then purified by column chromatography (silica, 6:3:2 n-hexane: ethanol: acetone) to give a brown solid (102 mg, 0.186 mmol, 52%): $R_f$= 0.48; LC-TOF-MS ES+, obs.=549 (14.006 min), calc.=548.69 [$(M)^+$, M = $C_{34}H_{36}N_4O_3$].

*(iii) Pyropheophorbide a (PyPh a).* Me-PyPh *a* (102 mg, 0.186 mmol) was dissolved in THF (25 mL) to which aqueous lithium hydroxide monohydrate (3 M, 10 mL) was added. This mixture was stirred under argon at room temperature for 18 h. Once complete, the solution was neutralised with addition of 3 M aqueous hydrochloric acid dropwise until pH 7 was reached according to universal indicator paper. The organic layer was then extracted with dichloromethane (3 x 50 mL), washed with water (3 x 100 mL) followed by brine (100 mL), then dried with magnesium sulfate, filtered and evaporated as before. This resulted in a black solid (71 mg, 0.138 mmol, 74%): TOF-MS ES+, obs. = 535.1, calc. = 534.66 [$(M + H)^+$, M = $C_{33}H_{34}N_4O_3$].

*(iv) Zinc-pyrochlorophyllide a (Zn-pyChl).* PyPh *a* (71 mg, 0.138 mmol) was dissolved in dichloromethane (35 mL) and saturated zinc acetate monohydrate in methanol (4 mL) was added to this. The solution was then refluxed at 35 °C under argon for 40 min, after which the solution was observed to changed colour form dark brown to dark green. The organic layer was then extracted with ethanol (40 mL) followed by washing with water (3 x 50 mL). Drying with magnesium sulfate, filtration and solvent removal were then carried out as before to give a blue-green solid (66 mg, 0.110 mmol, 80%).

*(v) Succinimidyl zinc-pyrochlorophyllide a (SC-Zn-pyChl).* Zn-pyChl (10 mg, $1.67 \times 10^{-5}$ mol) was mixed with DPTS (5 mg, $1.7 \times 10^{-5}$ mol), NHS (20 mg, 0.174 mmol) and crystalline EDC (22 mg, 0.115 mmol), followed by evacuation and backfilling with argon. The mixture was then dissolved in dry $CH_2Cl_2$ (36 mL) under an argon atmosphere, and the resulting solution

was then stirred for 16 h at room temperature under argon. Once complete, the solution was diluted with dichloromethane (50 mL) and washed with water (2 x 50 mL) and once more with saturated brine solution (50 mL). The organic layer was then dried with $MgSO_4$, filtered and evaporated as before to give a green solid (11 mg, $1.58 \times 10^{-5}$ mol, 95%).

*Analytical Data*

*Synthesis of cysteine methacrylate (CysMA)* [1]

$^1$H NMR (400 MHz, $D_2O$) *δ* (ppm)=1.86 (s, 3H), 2.60–3.15 (m, 6H), 3.74 (m, 1H), 3.84 (m, 1H), 4.08–4.43 (m, 4H), 5.67 (s, 1H), 6.09 (1H); $^{13}$C NMR (400 MHz, $D_2O$) *δ* (ppm) = 17.37, 26.41, 32.18, 33.95, 53.61, 65.27, 65.34, 66.90, 127.18, 135.66, 168.72, 172.75, 174.14; TOF MS LD$^+$: expected = 335.37 g·mol$^{-1}$, observed = 336.0 [$(M+H)^+$, M=$C_{13}H_{21}NO_7S$]; HPLC (30 min, 10% Acetonitrile in water (0.1% trifluoroacetic acid), detection = 225 nm) = 22.8 min, 90.0%.

*Extraction and modification of n-hydroxysuccinimidyl zinc-pyrochlorophyllide a*

$R_f$ =0.33 (6:3:1 hexane: ethanol: acetone); LC-TOF-MS ES+, obs. *m/z* = 872 (17.191 min), calc. = 871.22 [$(M + H)^+$, M=$C_{55}H_{74}N_4O_5$].

$^1$H NMR (400 MHz, $CDCl_3$) δ (ppm) = 0.09, -1.60 (br s, 1H, NH), 0.69-0.97 (m, pythyl CH-$CH_3$) 0.96-1.41 (m, pythyl $CH_2$), (m, pythyl vinyl, 2H) 1.82 (d, *J* = 6.29, 3H), 1.90 (m, 3H), 2.08 (m, 3H), 2.19, 2.36 (m, 2H), 2.50, 2.64 (m, 2H), 2.83 (br t, *J* = 5.39, 2H), 3.26 (s, 3H, methyl), 3.42 (s, 3H, methyl), 3.71 (br s, 3H, methyl), 3.71 (br s, 2H, $CH_2$-$CH_3$) 3.90 (s, 3H, $COOCH_3$), 4.23 (br d, *J* = 7.71 Hz, 1H) 4.31 (m, 2H, pythyl $CH_2$), 4.46 (m, 1H HC-$CH_3$), 4.50 (m, 3H, $CH_3$) 5.15 (m, 1H, pythyl vinyl), 5.14, 5.38 (m, multiple H, aggregation), 6.18 (d, *J* = 9.16, 1H, vinyl), 6.22 (s, 1H, C*H*-$COOCH_3$), 6.31 (d, *J* = 19.21 Hz, 1H, vinyl, cis), 8.02 (dd, *J* = 11.28, *J* = 5.97, 1H, vinyl, trans) 8.58 (s, 1 H, ring δ-H), 8.85 (s, 1H, ring α-H), 9.40 (s, 1H, ring β-H).

*Methyl pyropheophorbide a (Me-PyPh a).*

$^1$H NMR (400 MHz, $CDCl_3$) δ (ppm) = 0.13, -0.71 (br s, 2H, NH) 0.85-0.96 (m, aggregation), 1.19-1.39 (m, aggregation), 1.47 (s, 3H), 1.70 (t, *J* = 7.66 Hz, 3H), 1.89 (d, *J* = 5.77, 3H), 2.06 (m, 3H), 2.19, 2.33 (m, 2H), 2.59, 2.73 (m, 2H), 2.84 (br t, *J* = 5.95 Hz, 2H), 3.22 (s, 3H), 3.42 (s, 3H), 3.65 (s, $COOCH_3$, 3H), 3.66 (s, 3H), 3.67 (s, 2H), 3.70 (s, 3H),

4.31 (br d, *J* = 7.16 Hz, 1H), 4.51 (dd, *J* = 7.16 Hz, 1H), 5.21 (dd, *J* = 19.62 Hz, J = 42.55 Hz, 2H), 5.38 (m, multiple H, aggregation), 6.18 (d, *J* = 11.57 Hz, 1H, vinyl), 6.29 (d, *J* = 17.90, 1H, vinyl), 7.02 (s, 1H, cis), 7.99, (dd, *J* = 6.10 Hz, *J* = 11.45 Hz, 1H, vinyl, trans), 8.59 (s, 1H, ring δ-H), 9.37 (s, 1H, ring α-H), 9.48 (s, 1H, ring β-H).

*Pyropheophorbide a (PyPh a)*

$^{1}$H NMR (400 MHz, $CDCl_3$) δ (ppm) = 0.12, -1.73 (br s, 1H, NH), 0.89 (br dd, *J* = 6.61 Hz, 2H), 1.24 (d, *J* = 6.15 Hz, 3H), 1.25-1.39 (m, aggregation), 1.65 (t, *J* = 7.68 Hz, 3H), 1.83 (d, J = 7.09 Hz, 3H), 2.24, 2.36 (m, 2H), 2.65 (m, 2H), 3.15 (s, 3H), 3.38 (s, 3H), 3.06 (s, 3H), 3.59 (s, 3H), 4.06 (q, *J* = 6.15 Hz, 1H), 4.29 (br d, J = 9.22 Hz, 1H), 4.48 (br dd, *J* = 6.66, *J* = 7.43 Hz, 1H) 5.18 (q, *J* = 19.84 Hz, J = 41.50 Hz, 2H), 6.13 (d, *J* = 11.46 Hz, 1H, vinyl), 6.24 (d, *J* = 18.09 Hz, 1H, vinyl, cis), 7.91 (dd, *J* = 6.26 Hz, J = 11.46 Hz, 1H, vinyl, trans), 8.54 (s, 1H, ring δ-H), 9.26 (s, 1H, ring α-H), 9.36 (s, 1H, ring β-H).

*Zinc-pyrochlorophyllide a (Zn-pyChl a)*

$^{1}$H NMR (400 MHz, $CDCl_3$) *δ* (ppm) = 0.09 (s ??), 0.88 (br t, *J* = 7.46 Hz, 3H, methyl), 1.00 (t, *J* = 7.59 Hz, 3H), 1.05-1.48 (m, multiple H, aggregation), 1.53 (q, *J* = 6.70 Hz, 3H), 1.79 (d, *J* = 28.31 Hz, 3H, methyl), 2.05 (m, 2H, $\mathit{CH_2}$-$CH_2$-COOH), 2.32 (m, 2H, $CH_2$-$\mathit{CH_2}$-COOH), 2.83 (t, *J* = 5.68 Hz, 2H, $\mathit{CH_2}$-$CH_3$), 3.17 (s, 3H, methyl), 3.34 (s, 3H, methyl), 3.68 (s, 3H, methyl), 3.95 (d, *J* = 7.10, 2H), 5.17 (br m, 1H), 5.37 ( br m, 1H), 5.77 (dd, *J* =6.68 Hz, *J* = 10.96 Hz, 2H, vinyl), 6.39 (*J* =6.85 Hz, *J* = 10.57 Hz, 2H, vinyl), 7.00 (d, *J* = 7.23 Hz, 1H, vinyl, cis), 8.01 (m, 1H, vinyl, trans), 8.47 (br s, 1H, ring δ-H, 1H), 9.35 (br s, 1H, ring α-H), 9.55 (br s, 1H, ring β-H); TOF-MS ES+, obs. = 597.2, calc. = 598.024 $[(M - H)^+$, M = $C_{33}H_{32}N_4O_3Zn]$; IR ($CH_2Cl_2$ solvent, $cm^{-1}$) = 3500–2400 (broad band, O-H stretch), 3010 ($sp^2$ C-H stretch), 2926, 2858 ($sp^3$ C-H stretch), 1711 (C=O stretch), 1460 (aromatic C=C stretch), 1375 (C-O stretch); UV/Vis (dimethylformamide, nm) = 430 (Soret), 573 ($Q_x$), 656 ($Q_y$); HPLC (21 min, 75% Acetonitrile in water (0.1% trifluoracetic acid), detection=254 nm) = 12.4 min, 96.8%.

*Succinimidyl zinc-pyrochlorophyllide a (SC-Zn-pyChl a)*

TOF MS LD+: expected = 695.097 g $mol^{-1}$, observed = 693.5 $[(M–2H)^+$, M = $C_{37}H_{35}N_5O_5Zn]$.

$^{1}H$ NMR (400 MHz, $CDCl_3$) $\delta$ (ppm) = 0.09 (s ??), 0.88 (br q, $J$ = 2.65 Hz, $J$ = 6.68 Hz, 3H, methyl), 0.99 (t, $J$ = 7.52 Hz, 3H), 1.05-1.48 (m, multiple H, aggregation), 1.53 (q, $J$ = 6.6 Hz, $J$ = 6.89 Hz, 3H), 1.65 (d, $J$ = 16.07 Hz, 3H, methyl), 2.03 (m, 2H, $\mathit{CH_2}$-$CH_2$-COOH), 2.32 (m, 2H, $CH_2$-$\mathit{CH_2}$-COOH), 2.85 (d, $J$ = 21.23 Hz, 2H, $\mathit{CH_2}$-$CH_3$), 3.17 (s, 3H, methyl), 3.26 (s, 3H, methyl), 3.68 (s, 3H, methyl), 3.86 (d, $J$ = 6.82, 2H), 5.17 (br m, 1H), 5.32 (s, 4H, succinimidyl H), 5.37 ( br m, 1H), 5.76 (dd, $J$ =6.73 Hz, $J$ = 11.06 Hz, 2H, vinyl), 6.13 (m, multiple H, aggregation), 6.39 ($J$ =6.80 Hz, $J$ = 10.48 Hz, 2H, vinyl), 7.01 (d, $J$ = 7.33 Hz, 1H, vinyl, cis), 7.98 (q, $J$ = 6.18 Hz, $J$ = 11.48 Hz, 1H, vinyl, trans), 8.46 (s, 1H, ring δ-H, 1H), 9.24 (s, 1H, ring α-H), 9.41 (s, 1H, ring β-H); TOF MS LD+: expected = 695.097 g $mol^{-1}$, observed = 693.5 [$(M–2H)^+$, M = $C_{37}H_{35}N_5O_5Zn$]; FTIR (solid state, $cm^{-1}$) 3352 (N-H stretch), 3189 ($sp^2$ C-H stretch), 2959, 2921 (sharp, $sp^2$ C-H stretch), 2851 (sharp, $sp^3$ C-H stretch), 2274 (N=C=O stretch), 1779 (sharp, C=O stretch), 1729 (sharp, C=O stretch, amide), 1661 (C=C stretch), 1535 (N-O stretch), 1375 (C-O stretch), 1200 (sharp, C-O/C-N stretch); UV/Vis (dimethylformamide, nm) = 431 (Soret), 573 ($Q_x$), 658 ($Q_y$).

SURFACE-INITIATED POLYMERISATION

*Grafting of PCysMA brushes to gold substrates by ARGET-ATRP*

2,2'-Bipyridyl (≥ 99%), bis[2-(2-bromoisobutyryloxy)undecyl] disulfide (DTBU) (97%), Copper(II) chloride (99%) and L-ascorbic acid (reagent grade, ≥98%) were obtained from Sigma-Aldrich.

Substrates were cut from the coverslips coated with evaporated Cr/Au films, rinsed with ethanol and dried with nitrogen. The slides were immersed in a 2 mM solution of bis[2-(2-bromoisobutyryloxy)undecyl] disulfide (DTBU, the ATRP initiator) in nitrogen-degassed ethanol for 24 h to form a bromine-terminated initiator monolayer.

For the grafting of reduced-density films, bis[2-(2-bromoisobutyryloxy)undecyl] disulfide was mixed with 1-undecanethiol and dissolved in nitrogen-degassed ethanol to give a total concentration of 2 mM.

The slides were stored in the thiol solution for at least 24 h in a fridge at temperature of 4 °C until further use. When needed, the slides were rinsed thoroughly with ethanol and dried with nitrogen.

For the polymerisation reactions, solutions of CysMA (750 mg, 2.231 mmol, 4 mL H2O, 0.56 M), Copper(II) chloride ($CuCl_2$) (14.6 mg, 0.109 mmol, 5 mL $H_2O$), 2,2'-bipy (38.8 mg, 0.248 mmol, 5 mL ethanol) and l-ascorbic acid (100 mg, 0.568 mmol, 10 mL $H_2O$, 56.8 mM). The $CuCl_2$ and 2,2'-bipy solution were mixed together to form the vivid blue $Cu(bipy)_2Cl_2$ complex solution (10 mL, [CuCl2] = 10.9 mM, [2,2'-bipy] = 24.8 mM). To the CysMA solution, the l-ascorbic acid solution (0.18 mL, $1.02 \times 10^{-5}$ mol) was added first, followed by the Cu/bipy solution (0.35 mL, n(Cu) = $3.82 \times 10^{-6}$ mol). The solution was then gently shaken to ensure complete mixing and left for 7 - 10 min, by which time a brown colour had formed in solution, indicating the generation of active Cu(I) complexes.

A sample of gold/initiator substrate was then introduced to the solution for polymer grafting. Once the desired polymerization time was reached, the sample was withdrawn from the solution and washed well with deionised water followed by ethanol. Samples were stored in ethanol in a fridge at the temperature of 4°C until required. The same procedure was used both for unpatterned gold substrates and plasmonic arrays of gold nanostructures.

*Attachment of succinimydyl zinc-pyrochlorophyllide a to solvated PCysMA brushes*

N-Hydroxy succinimidyl ester derivatives of chlorophyll (Chl) were either used straight after synthesis or stored under dry argon at –20 °C to prevent hydrolysis before use. A 1 mM solution of Chl was made by dissolving it in DMF:H2O(1:3) mixture. Once dissolved, PCysMA samples were immersed in the chlorophyll solution and functionalisation allowed to occur for 16 - 18 h. Once complete, the samples were washed with DMF followed by deionised water. This same procedure was used for both continuous gold substrates and plasmonic arrays of gold nanostructures. Samples were stored in ethanol in a fridge at the temperature of 4 °C until required.

## CHARACTERISATION OF PLEXCITONIC COMPLEXES

*X-ray photoelectron spectroscopy (XPS)*

Mixed self-assembled monolayers formed by the adsorption of thiolates from mixtures of bis[2-(2-bromoisobutyryloxy)undecyl] disulfide and 1-undecanethiol were characterized by XPS. Measurements were performed using a Kratos Supra X-ray photoelectron spectrometer (Kratos Analytical, Manchester, UK) with a monochromated Al Kα x-ray source (1486 eV). The area of analysis was 300 x 700 μm. Survey spectra were acquired at pass energy of 160

eV. High-resolution C 1s, N 1s, O 1s, Au 4f, Br 3d scans were collected over appropriate energy ranges at 20 eV pass energy, with 0.1 eV intervals. All XPS spectra were analyzed and curve-fitted using the Casa XPS software and were corrected relative to the C 1s signal at a binding energy of 285.0 eV. Measurements were repeated at least three times, and errors quoted in numerical data are the standard deviations.

While the C1s spectrum of undecanethiol is dominated by a large hydrocarbon peak at 285 eV, the C1s spectra of bromoisobutyryl-functionalised undecanethiolate (BIBUDT) SAMs exhibit peaks at 286.6 eV (attributed to the combined contributions of carbon atoms adjacent to oxygen and bromine) and 289.2 eV (carboxylate carbon). Analysis of XPS spectra also enables confirmation of covalent attachment of N-hydroxysuccinimidyl ester derivatives of chlorophyll a to surface-grafted PCysMA (Figure S2). In the C1s region, binding of chlorophyll is accompanied by an increase in the (C–C–C) component at 285.0 eV, because each chlorophyll contains a large number of carbon atoms bound only to other carbon atoms and hydrogen. The component at 286.6 eV is slightly reduced in size because although each of the four tetrapyrrole units contains equal numbers of hydrocarbon atoms and carbon atoms bonded to nitrogen, there are an additional 10 carbon atoms in pendant methyl groups that increase the area of the feature at 285.0 eV.

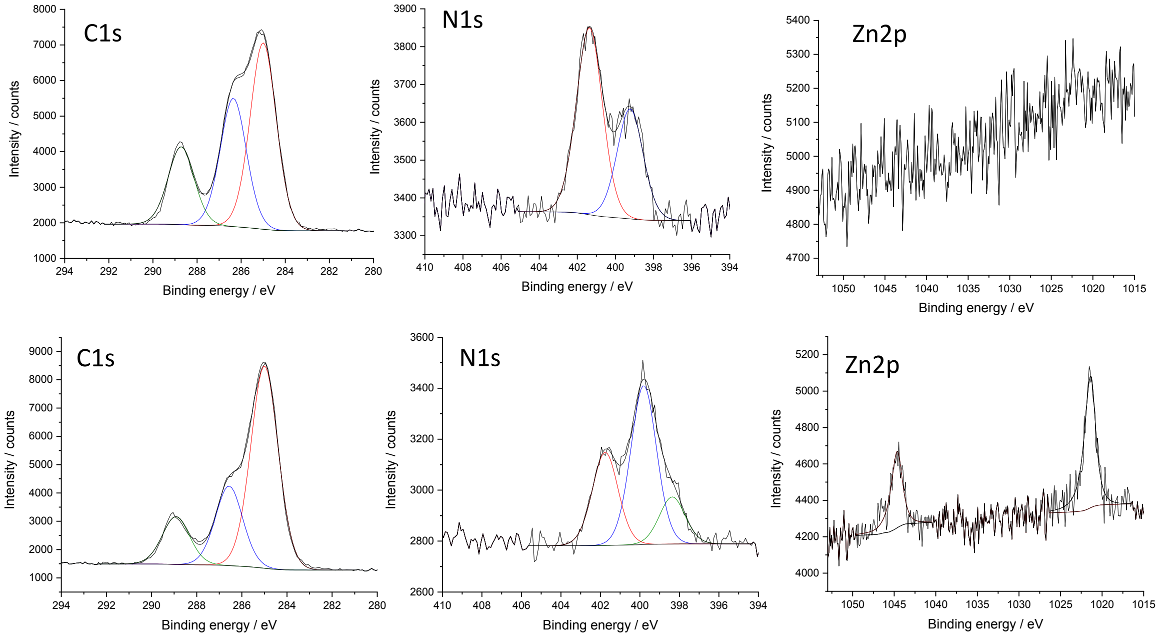


**Figure S2**. XPS C1s, N1s and Zn2p spectra before (top) and after (bottom) reaction of grafted PCysMA films with N-hydroxysuccinimidyl ester derivatives of chlorophyll a.

The N1s region displays marked changes following attachment of N-hydroxysuccinimidyl ester derivatives of chlorophyll a. PCysMA exhibits two N1s environments at 399.2 and 401.4 eV, corresponding to $-\underline{N}H_2$ and $-\underline{N}H_3^+$, respectively. However, after binding of chlorophyll a, the strongest peak observed in the N1s region is at 399.8 eV, together with a small peak at 398.4, both attributed to nitrogen in the tetrapyrrole units.

Finally, Zn is undetectable in the XPS spectrum of as-prepared grafted PCysMA films but is observed in the spectrum of chlorophyll-derivatised films because $Zn^{2+}$ is coordinated at the centre of the tetrapyrrole structure of chlorophyll a.

*UV-vis spectroscopy*

UV-visible absorption spectra at normal incidence were recorded in both air and liquids using a Cary50 spectrophotometer (Agilent Technologies, USA). The wavelength scan range was 480 – 900 nm (unless otherwise stated). The samples were fixed in a home-made PTFE holder enabling absorption measurements of the same spot on the sample during all experimental stages. During the measurements in liquid, the holder with a sample was placed in a standard quartz cuvette (10 mm transmitted path length).

Before thermal measurements, the samples were soaked in a stainless steel cell with two quartz windows filled with a solvent at room temperature (20 °C) for at least 45 min. The following solvents were used to investigate the response of the antenna complexes to pH and temperature modification: deionized water (pH 6.8 ± 0. 2), DMF:H2O (1:3) mix (pH 6.7 ± 0.2), DMF:PBS (1:3) mix (pH 7.9 ± 0.2), DMF:PBS (1:1) mix (pH 8.7 ± 0.2), 0.1M HCl pH (1.0 ± 0.20), 1M NaOH (pH 12.0 ± 0.2). A thermal cycle was applied by heating the solution through a copper plate underneath the cell. The temperature of the liquid media was measured continuously with a CAL9000 controller (CAL, USA) equipped with Fe-CuNi thermocouple immersed in the sample cell. A precision of the measurement was ±0.01 °C.

*Spectroscopic ellipsometry*

Ellipsometric $\Psi(\lambda)$ and $\Delta(\lambda)$ spectra of continuous Cr/Au thin films functionalized with SAMs of BIBUDT, BIBUDT:UDT mixtures, grafted with PCysMA brushes, and PCysMA brushes derivatized with Chl, were collected with an Alpha-SE (J. A. Woollam Co., Inc., USA) ellipsometer operating in a spectral range of 370-1000 nm at the incident angle of 75°. All data were acquired and modelled using CompleteEASE (v.6.6) software. The oval-shape beam was

in the size of the ca. 1.46 x 0.35 cm. Measurements were carried out in the air and each sample was measured at 3 locations to obtain an average.

To evaluate the thickness of dry-state PCysMA brushes derivatized with chlorophyll grown on continuous gold surface, an optical multilayer-box-model was applied (see Table S2). Values for optical constants *n* and *k* of glass, chromium and gold were taken from the standard material library.

A Cauchy dispersion function ($n = A_n + \frac{B_n}{\lambda^2} + \frac{C_n}{\lambda^4}$ and $k = 0$, where *A, B*, and *C* are constants, *A* sets amplitude, *B* and *C* give dispersion shape) for the non-absorbing initiator-filler (i.e. BIBUDT:UDT) layer at a different ratio was used for modelling the adsorbed SAMs layer of initiator and poly(cysteine methacrylate) brushes. Refractive indices *n* of the initiator-filler mixtures used in this model, were measured previously with the Abbe refractometer. BIBUDT:UDT film thicknesses on continuous gold surface were fitted taking into account the previously measured Cr/Au layer thickness.

For the PCysMA brush layer, a Cauchy relation in combination with an effective medium approach (EMA) according to Bruggeman was used to describe the dependence of the refractive index *n* on the wavelength for this non-absorbing polymer. The refractive index of PCysMA was measured with the Abbe refractometer and set to ca.1.53. The second material in EMA model was either void (before derivatization of PCysMA brushes with chlorophyll) or chlorophyll (after derivatization). During modelling, the thickness and the optical constants of the under-lying initiator layer were fixed to the values determined to avoid parameter correlations.

The volume fraction of chlorophyll inside the PCysMA brush layer was modelled by an EMA approach. Dispersion relations for chlorophyll were determinate previously using B-spline function. Uniqueness of fit values with the absence of correlated parameters in the fitting process were checked for every fitted parameter.

**Table S2.** The model used for spectroscopic ellipsometry data fitting of polymer brushes derivatized with chlorophyll grown on continuous gold film.

| *Layer* | *Material* | *Fitted parameters* |
|---|---|---|
| Layer #4 | EMA:<br>Material #1: Cauchy (PCysMA brushes)<br>Material #2: Chlorophyll | thickness *d* of the EMA layer in nm<br>Fraction of Material#2<br>Depolarization |
| Layer #3 | Cauchy_Wvl (initiator) | thickness *d* of the adsorbed SAMs of initiator in nm<br>Refractive index *n* and parameters $B_n$=0.01 and $C_n$=0 were fixed during the fitting. |
| Layer #2 | Au | thickness *d* of the evaporated gold film in nm |
| Layer #1 | Cr | thickness *d* of the evaporated chromium film in nm |
| Substrate | BK7 glass | |

To measure the thickness of a PCysMA brushes in liquid, a variable angle spectroscopic ellipsometer M-2000VI (J.A. Woollam Co., Inc., USA) operating over a range of wavelength between 370 - 1670 nm was used in conjunction with a home-made PTFE liquid cell. Incident angle was fixed at 70° to achieve irradiation of the sample perpendicular to the cuvette quartz windows, such that the light beam could be transmitted to the liquid without significant refraction. The sample was placed at the bottom of the cell and the beam aligned so that it entered and exited cleanly through the windows and was wholly reflected from the sample surface. The volume of the PTFE liquid cell used was 5 $cm^3$.

SPECTROSCOPY OF PIGMENT-POLYMER COMPLEXES ON GLASS

As a control, to determine whether changes in the exciton energy and linewidth might influence plasmon-exciton coupling, we acquired absorption spectra for pigment-polymer complexes grafted onto glass surfaces. To produce grafted polymers displaying a range of confirmations similar to those of the polymers grafted to gold nanostructures, we grew PCysMA from brominated initiators (aminopropyl triethoxy silane films derivatised by reaction with bromo isobutyryl bromide, following well-established methods) the density of which was varied by exposure of the substrates to a range of UV doses ($\lambda$ = 244 nm), following work published previously from this laboratory.[3] UV exposure causes photolysis of the C-Br bond, thus enabling controlled removal of the initiator leading to systematically varying grafting densities. An exposure of ~ 6 J cm$^{-2}$ is expected to yield substantial removal of the initiator, such that the polymer conformation is a mushroom, equivalent to the conformations produced at the lowest initiator densities reported in the main manuscript.

Figure S3 shows a variety of absorption spectra acquired for these materials. It can be seen from Figure S3a that there is no significant change in either the position or the line width of the Qy peak at 669 nm as a function of the polymer morphology. Figure S3b compares spectra for fully-dense brushes with polymerisation times of 10 and 30 min. Again, there appears to be no significant change in the energy or linewidth of the transition.

These data suggest strongly that variations in the Qy energy and linewidth do not contribute to the changes in plasmon-exciton coupling described in the main manuscript.

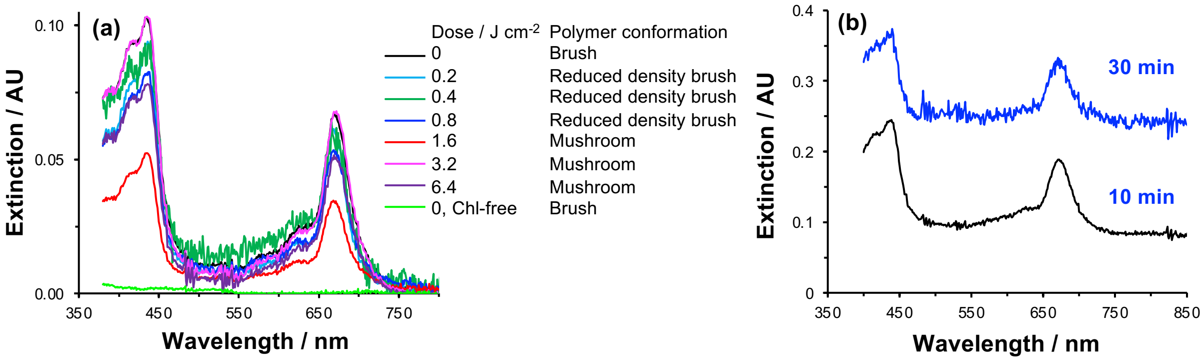


**Figure S3.** (a) Variation in the absorption spectra of pigment-polymer complexes formed on glass as a function of the UV dose used to systematically cleave Br initiators from the surface prior to ATRP. (b) Comparison of absorption spectra for Chl-functionalised fully-dense brushes for polymerisation times of 10 and 30 min.

MODELLING OF STRONG PLASMON-EXCITON COUPLING

Plasmon-exciton coupling in plexcitonic complexes was modelled as coupled oscillators, following the method described in our previous work.[4-6] In particular, we modelled a system consisting of a broad mode with resonance frequency $\omega_b$ and damping $\gamma_b$ coupled to a second narrow mode with resonance frequency $\omega_d$ and damping $\gamma_d$.[7, 8] The coupling between the oscillators is $g$ and the highly damped oscillator is driven by an external harmonic force with amplitude $fe^{i\omega t}$. In the present case, the broad, highly damped mode is the LSPR and the narrow line width mode is the exciton. The equations of motion are:

$$\ddot{x}_b + \gamma_b \dot{x}_b + \omega_b^2 x_b + g x_d = f e^{i\omega t} \quad (1)$$

$$\ddot{x}_d + \gamma_d \dot{x}_d + \omega_d^2 x_d + g x_b = 0 \quad (2)$$

For harmonic oscillators these equations yield solutions $x_b = c_b e^{i\omega t}$ and $x_d = c_d e^{i\omega t}$, where the coefficients $c_b$ and $c_d$ can be solved analytically. The extinction becomes proportional to the imaginary part of

$$\alpha(\omega) \propto \frac{1}{\tilde{f}(\omega) + i\omega\tilde{\gamma}(\omega)} \quad (3)$$

where

$$\tilde{f}(\omega) = \omega_b^2 - \omega^2 - \frac{g^2(\omega_d^2 - \omega^2)}{(\omega_d^2 - \omega^2)^2 + \gamma_d^2\omega^2} \quad (4)$$

and

$$\tilde{\gamma}(\omega) = \gamma_b + \frac{g^2\gamma_d}{(\omega_d^2 - \omega^2)^2 + \gamma_d^2\omega^2} \quad (5)$$

Using these it is possible to model the behaviour observed in the experimental system. We scale the equations to have units of energy rather than frequency. Thus, $G = \hbar^2 g$ is the scaled coupling which has the dimensions of energy squared. Similarly, the energy of the exciton $E_{\mathrm{mol}}$ corresponds to $\omega_d$ and the energy of the localised surface plasmon resonance $E_{\mathrm{LSPR}}$ corresponds

to $\omega_b$. The quantity $E_C = G/E_{LSPR}$ is approximately equal to the splitting in the coupled harmonic oscillator model when the two oscillators are at resonance with each other.[9]

Several criteria are used for determining whether a system is in the strong coupling regime[9]. In order for a normal mode splitting (Rabi splitting) at resonance to be visible, in the case where the linewidths of the modes are similar, the coupling energy $E_C$ should be roughly the same as or larger than the average of the linewidths of the two modes/oscillators (modes 1 and 2). However, this is only an overall criterion since the visibility also depends on the shapes of the linewidths. In case of one large and one small linewidth, a double-peak structure (on and slightly off resonance) becomes more easily visible and sometimes the condition $E_C \geq \sqrt{\gamma_1\gamma_2}$ is used as a guideline. In that case it is important to consider the fundamental criterion of strong coupling, namely that $E_C \geq \frac{1}{2}(\gamma_1 - \gamma_2)$. This guarantees that the term giving the splitting between two modes at resonance, $\sqrt{E_C^2 - \frac{1}{4}(\gamma_1 - \gamma_2)^2}$, stays real and indeed two different normal modes exist. In our case $\gamma_{mol}$ is ca. 0.1 eV, much smaller than $\gamma_P$ which can be ~0.5 eV. Two-peak structures and a shift of the peaks with coverage (exciton density) are obviously visible in our data (Figure 5 in the main manuscript). While these indicate strong coupling, it is important to check that the fundamental criterion is satisfied. Films with polymerisation times ≥ 10 min and reduced grafting densities, fulfil the criterion $E_C \geq \frac{1}{2}(\gamma_1 - \gamma_2)$, as discussed in the main text.

REFERENCES

1. A. Tsargorodska, O. El Zubir, B. Darroch, M. l. L. Cartron, T. Basova, C. N. Hunter, A. V. Nabok and G. J. Leggett, *ACS Nano*, 2014, **8**, 7858-7869.
2. A. M. Alswieleh, N. Cheng, I. Canton, B. Ustbas, X. Xue, V. Ladmiral, S. Xia, R. E. Ducker, O. El Zubir, M. L. Cartron, C. N. Hunter, G. J. Leggett and S. P. Armes, *Journal of the American Chemical Society*, 2014, **136**, 9404-9413.
3. O. Al-Jaf, A. Alswieleh, S. P. Armes and G. J. Leggett, *Soft Matter*, 2017, **13**, 2075-2084.
4. A. Tsargorodska, M. L. Cartron, C. Vasilev, G. Kodali, O. A. Mass, J. J. Baumberg, P. L. Dutton, C. N. Hunter, P. Törmä and G. J. Leggett, *Nano Letters*, 2016, **16**, 6850–6856.
5. A. Lishchuk, G. Kodali, J. A. Mancini, M. Broadbent, B. Darroch, O. A. Mass, A. Nabok, P. L. Dutton, C. N. Hunter, P. Törmä and G. J. Leggett, *Nanoscale*, 2018, **10**, 13064-13073.

6. A. Lishchuk, C. Vasilev, M. P. Johnson, C. N. Hunter, P. Törmä and G. J. Leggett, *Faraday Discussions*, 2019, DOI: 10.1039/C8FD00241J.
7. B. Gallinet and O. J. F. Martin, *Physical Review B*, 2011, **83**, 235427.
8. A. I. Väkeväinen, R. J. Moerland, H. T. Rekola, A. P. Eskelinen, J. P. Martikainen, D. H. Kim and P. Törmä, *Nano Letters*, 2014, **14**, 1721-1727.
9. P. Törmä and W. L. Barnes, *Reports on Progress in Physics*, 2015, **78**, 013901.
